# Derivation of totipotent-like stem cells with blastocyst-like structure forming potential

**DOI:** 10.1038/s41422-022-00668-0

**Published:** 2022-05-04

**Authors:** Yaxing Xu, Jingru Zhao, Yixuan Ren, Xuyang Wang, Yulin Lyu, Bingqing Xie, Yiming Sun, Xiandun Yuan, Haiyin Liu, Weifeng Yang, Yenan Fu, Yu Yu, Yinan Liu, Rong Mu, Cheng Li, Jun Xu, Hongkui Deng

**Affiliations:** 1 MOE Engineering Research Center of Regenerative Medicine, School of Basic Medical Sciences, State Key Laboratory of Natural and Biomimetic Drugs, Peking University Health Science Center and the MOE Key Laboratory of Cell Proliferation and Differentiation, College of Life Sciences, Peking-Tsinghua Center for Life Sciences, Peking University, Beijing, China; 2 School of Life Sciences, Center for Bioinformatics, Center for Statistical Science, Peking University, Beijing, China; 3 Department of Rheumatology and Immunology, Peking University Third Hospital, Beijing, China; 4 Beijing Vitalstar Biotechnology Co., Ltd, Beijing, China; 5 Program for Cancer and Cell Biology, Department of Human Anatomy, Histology and Embryology, School of Basic Medical Sciences, PKU International Cancer Institute; MOE Key Laboratory of Carcinogenesis and Translational Research and State Key Laboratory of Natural and Biomimetic Drugs, Peking University Health Science Center, Beijing, China; 6 Department of Cell Biology, School of Basic Medical Sciences, Peking University Stem Cell Research Center, Peking University Health Science Center, Peking University, Beijing, China

**Keywords:** Totipotent stem cells, Self-renewal

## Abstract

It is challenging to derive totipotent stem cells in vitro that functionally and molecularly resemble cells from totipotent embryos. Here, we report that a chemical cocktail enables the derivation of totipotent-like stem cells, designated as totipotent potential stem (TPS) cells, from 2-cell mouse embryos and extended pluripotent stem cells, and that these TPS cells can be stably maintained long term in vitro. TPS cells shared features with 2-cell mouse embryos in terms of totipotency markers, transcriptome, chromatin accessibility and DNA methylation patterns. In vivo chimera formation assays show that these cells have embryonic and extraembryonic developmental potentials at the single-cell level. Moreover, TPS cells can be induced into blastocyst-like structures resembling preimplantation mouse blastocysts. Mechanistically, inhibition of HDAC1/2 and DOT1L activity and activation of RARγ signaling are important for inducing and maintaining totipotent features of TPS cells. Our study opens up a new path toward fully capturing totipotent stem cells in vitro.

## Introduction

During development, early-stage blastomeres are totipotent cells that have the potential to generate an entire individual, including both embryonic and extraembryonic components, at the single-cell level.^1,2^ The totipotent potential of early-stage blastomeres is gradually restricted at the blastocyst stage, when differentiation into epiblast, trophectoderm and primitive endoderm occurs.^3–5^ Different self-renewing stem cells, including pluripotent stem cells, trophoblast stem cells and extraembryonic endoderm cells, can be derived from the blastocyst,^6–9^ which preserve the developmental potentials of epiblast, trophectoderm and primitive endoderm, respectively. Importantly, the developmental potentials of these stem cell types are lineage restricted in that they have difficulties in crossing the embryonic and extraembryonic lineage boundaries especially in vivo.^10–12^ Compared to these blastocyst-derived stem cell types, totipotent embryonic cells harbor the greatest developmental potency, and there has been great scientific interest in capturing such stem cells from totipotent embryos in vitro,^13^ whose unrestricted potency would have broad applications for stem cell biology and regenerative medicine.

Previous studies have shown that a rare 2-cell embryo (2C)-like subpopulation exists in mouse embryonic stem (ES) cell cultures that express 2-cell blastomere molecular markers and have embryonic and extraembryonic developmental potentials.^14,15^ The major limitation of 2C-like cells is that they are only a minor population from mouse ES cell cultures and reside in a transient intermediate state, and thus these cells cannot be stabilized in vitro.^13^ In recent years, our group and others have shown that small molecule combinations can expand the developmental potentials of pluripotent stem cells toward extraembryonic lineages.^16,17^ Importantly, extended pluripotent stem (EPS) cells can be induced into blastocyst-like structures.^18^ Despite their expanded developmental potentials, these cells are still transcriptomically distinct from 2-cell embryos.^19^ In addition, their extraembryonic differentiation potentials are still limited compared to totipotent embryos.^19^ Therefore, it remains a major challenge to capture and maintain bona fide totipotent stem cells in vitro. In principle, such stem cells should be derived directly from totipotent embryos, which represents the gold criterion to capture authentic totipotency.

In this study, through chemical screening and combination, we identified a chemical cocktail that can directly establish totipotent-like stem cells from 2-cell embryos in vitro, as well as convert from EPS cells. The induced totipotent-like stem cells can be stably maintained long term in vitro, with molecular features resembling 2–4-cell blastomeres. Moreover, they can generate both embryonic and extraembryonic lineages in vivo at the single-cell level and form blastocyst-like structures in vitro.

## Results

### Identification of a chemical cocktail that can establish totipotent-like stem cells in vitro

To identify small molecules that can induce totipotent features in mouse EPS cells, we used cell lines carrying a MERVL-tdTomato or a Zscan4-Emerald GFP reporter. As a positive control, *Dux* overexpression was induced in these reporter cells (Supplementary information, Fig. S1a–c). N2B27 medium was used for screening and Wnt signaling agonist CHIR 99021 was also added to promote cell proliferation. In the primary screen, we focused on small molecules that can increase the percentage of Zscan4-Emerald GFP^+^ cells, and identified > 50 primary hits. These candidates were further evaluated using MERVL-tdTomato reporter cells and small molecules that can also induce MERVL-tdTomato expression were selected for further determination of their optimal dosage in activating Zscan4-Emerald and MERVL-tdTomato expression without inducing significant toxicity. After the titration, we found that CD1530 was most effective in inducing Zscan4-GFP and MERVL-tdTomato expression (Supplementary information, Fig. S1d–f). Therefore, we tested the effect of CD1530 combined with other selected candidates on induction of totipotency marker genes, and found that VPA and EPZ004777 showed a synergistic effect with CD1530 (Supplementary information, Fig. S1g). We also tested whether CHIR 99021 could be omitted from the cocktail, and found that cell proliferation was greatly reduced under such condition during the conversion (Supplementary information, Fig. S1h, i). Therefore, CHIR 99021 was included in the chemical cocktail. Collectively, we identified a chemical cocktail inducing totipotency, which include CD1530, VPA, EPZ004777 and CHIR 99021 (CPEC condition).

**Fig. 1:**
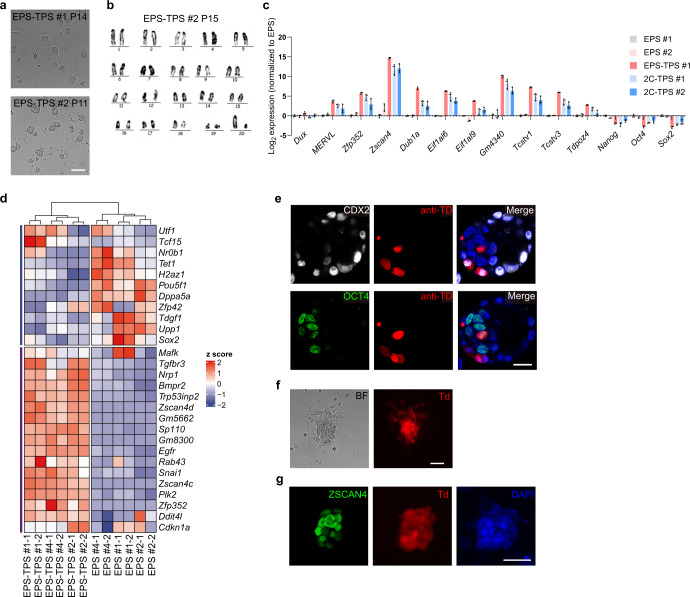
Identification of a chemical cocktail that induce totipotent stem-like cells in vitro. **a** Representative images showing induction of TPS cells from EPS cells under the CPEC condition. Scale bar, 100 μm. Similar images were obtained in at least 5 independent experiments. **b** Karyotype analysis of TPS cells after long-term culturing under the CPEC condition. Similar images were obtained in 3 independent experiments. **c** qPCR analysis of expression of totipotency and pluripotency marker genes in TPS cells converted from EPS cells (EPS-TPS #1) and TPS cells derived from 2-cell embryos (2C-TPS #1 and #2). *n* = 3 biological replicates. Similar results were obtained in at least 2 independent experiments. Relative expressions were normalized to EPS #1. **d** Heatmap showing the relative expression of representative totipotency and pluripotency genes in TPS and EPS cells. **e** Representative immunofluorescent analysis showing expression of trophectoderm (CDX2) and epiblast (OCT4) markers in TPS derivatives in the chimeric blastocysts. These images showed different focal planes of the same embryo. Scale bar, 20 μm. anti-TD, immuno-staining of tdTomato protein. Similar images were obtained in at least 2 independent experiments. **f** Representative morphology of CPEC-treated outgrowth derived from 2-cell embryos expressing tdTomato. Scale bar, 100 μm. BF, bright field. Td, tdTomato. Similar images were obtained in at least 2 independent experiments. **g** Representative immunofluorescent analysis of ZSCAN4 expression in TPS cells derived from 2-cell embryos expressing tdTomato. Scale bar, 50 μm. Td, endogenous tdTomato. Similar images were obtained in at least 2 independent experiments.

We further analyzed whether the cells cultured under the CPEC condition could be stably maintained in vitro as well as preserve the totipotent features. CPEC-cultured cells can be maintained and passaged for > 10 passages with normal karyotype (Fig. 1a, b). We also found that the average length of telomere is longer in these cells compared to those in ES and EPS cells (Supplementary information, Fig. S1j). After long-term culture (> 10 passages), CPEC-treated cells still maintained the expression of totipotency marker genes such as *Zscan4*, *Zfp352*, *Tcstv1*, *Tcstv3* and *MERVL* (Fig. 1c), and the protein expression of ZSCAN4 could be detected in these cells (Supplementary information, Fig. S1k). Meanwhile, the expression of pluripotency marker genes, including *Oct4*, *Nanog* and *Sox2*, was significantly downregulated in these cells (Fig. 1c). We also compared the expression of totipotency marker genes in CPEC-treated cells at different passages, and found that cells after passage 5 showed a relatively stable upregulation of totipotency marker genes, implying that at least 5 passages are required for obtaining stable totipotent features in the CPEC-treated cells (Supplementary information, Fig. S1l). Consistent with quantitative PCR (qPCR) analysis, bulk RNA-sequencing (RNA-seq) analysis also confirmed the upregulation of multiple totipotency marker genes as well as downregulation of multiple pluripotent marker genes (Fig. 1d).

To analyze the stability of cells expressing totipotency marker genes, we also enriched these cells using a Zscan4-Emerald GFP reporter and cultured them for 5 passages in vitro. CPEC-treated cells proliferated well after the sorting, whereas the purified cells cultured without CPEC treatment grew poorly (Supplementary information, Fig. S1m). Further qPCR analysis showed that the expression of totipotency marker genes could be maintained in the CPEC-treated cells after purification and culturing (Supplementary information, Fig. S1n). We also tested whether the CPEC condition could induce totipotency marker gene expression in mouse ES cells, and found upregulation of multiple totipotency marker genes in CPEC-treated ES cells after 5 passages (Supplementary information, Fig. S1o). Collectively, these results indicated that CPEC-treated cells acquired totipotent molecular features and were distinct from conventional pluripotent cell types.

Because the CPEC-treated cells exhibited totipotent molecular features, we further explored whether they possessed extraembryonic developmental potentials in vitro. To this end, CPEC-treated cells were cultured and passaged in a mouse trophoblast stem (TS) cell culture medium. After 2–4 passages, multiple flat mouse TS-like colonies emerged, which could be further passaged in mouse TS medium. Immunofluorescent analysis showed that these TS-like cells expressed TS markers including EOMES, TFAP2C CDX2, and SOX2 (Supplementary information, Fig. S2a), suggesting that they acquired the identity of trophoblast lineages. Consistent with these results, CPEC-treated cells could also be induced into cells expressing markers of primitive endoderm (PE) upon culturing in the medium that induce PE differentiation (Supplementary information, Fig. S2b).

To evaluate the developmental potentials of CPEC-treated cells, we performed in vitro chimera formation experiments. CPEC-treated cells that continuously expressed tdTomato were injected into 8-cell mouse embryos which were cultured in vitro for 48 h. Immunofluorescent analysis of the chimeric mouse embryos showed that derivatives from CPEC-treated cells expressed markers of trophectoderm (TFAP2C, CK8, CDX2, EOMES), epiblast (OCT4) and primitive endoderm (PDGFRα) (Fig. 1e; Supplementary information, Fig. S3). Collectively, these results suggest that cells cultured under this new condition could be maintained long term while preserving the ability to express totipotency marker genes and bi-developmental potentials, therefore we designated these totipotent-like stem cells as totipotent potential stem (TPS) cells.

We further explored whether TPS cells can be directly derived from 2-cell embryos. To this end, 2-cell mouse embryos without zonal pellucida were seeded on feeder cells and cultured under the CPEC condition. We found that some embryos transformed into compact cell clumps under the CPEC condition followed by the emergence of outgrowth (Fig. 1f). The outgrowth can be further passaged, resulting in the subsequent emergence of domed TPS colonies, which showed expression of ZSCAN4 (Fig. 1g). Similar to TPS cells that were induced from EPS cells, TPS cells established from 2-cell embryos also expressed totipotency marker genes (Fig. 1c). These results indicate that the CPEC condition enables the derivation of TPS cells directly from 2-cell mouse embryos.

### TPS cells share transcriptomic features with 2-cell blastomeres

To explore the molecular features of TPS cells, we analyzed the transcriptomic differences between TPS cells and EPS cells using bulk RNA-seq data. Hierarchical clustering analysis showed that the global transcriptome of TPS cells was distinct from that of EPS cells (Supplementary information, Fig. S4a). Identification of genes that were differently expressed between TPS cells and EPS cells showed the presence of representative totipotency marker genes, such as *Gm5662*, *Zscan4c* and *Zfp352* (Supplementary information, Fig. S4b). We further performed Gene Ontology (GO) analysis and found that the differentially expressed genes (DEGs) are majorly involved in the regulation of development (Supplementary information, Fig. S4c).

Next, we analyzed the expression levels of these DEGs in early preimplantation development. Notably, the genes upregulated in TPS cells were also highly expressed at the 2C-embryo stage, and their expression was gradually decreased at the blastocyst stage (Supplementary information, Fig. S4d). On the contrary, the genes downregulated in TPS cells were more abundantly expressed at the blastocyst stage but not the 2C-embryo stage (Supplementary information, Fig. S4d). We also identified totipotency signatures including 2399 genes that are upregulated in 2-cell totipotent embryos in comparison with epiblast cells from blastocysts using published single-cell RNA-seq data of preimplantation mouse embryos.^20^ Among these genes, 1585 genes were significantly upregulated in TPS cells compared to EPS cells (Supplementary information, Fig. S4e). These data suggest that TPS cells possess transcriptomic features that are specific to 2-cell embryos.

**Fig. 2:**
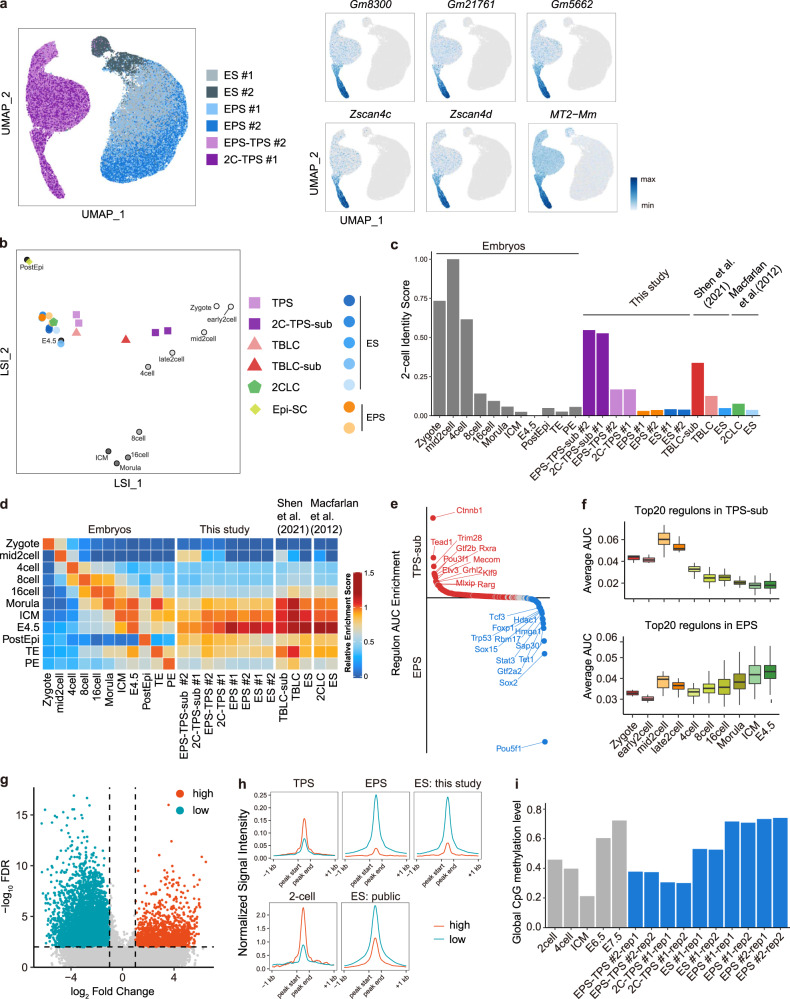
TPS cells share transcriptomic and epigenetic features with 2-cell blastomere. **a** UMAP plot showing the expression of representative totipotency marker genes in TPS, ES and EPS cells at the single-cell level. Different cell types are indicated using different colors. **b** Latent semantic indexing (LSI) analysis comparing developmental progression from zygote to postimplantation (E5.25–E7.5) epiblast with TPS cells, ES cells, EPS cells, TBLCs and spontaneous 2C-like cells. 2C-TPS-sub, TPS 2C-subpopulation; TBLC-sup, subpopulation from TBLCs; 2CLC, spontaneous 2C-like cells. The detailed method for constructing the mouse embryonic development trajectory is provided in Materials and methods section. Sequencing data of different stem cell types are from GSE33923,^14^ GSE168728,^21^ GSE74155,^66^ and GSE145609.^19^ **c** Quadratic programming-based deconvolution analysis showing the transcriptomic similarity between 2-cell embryo and different in vitro cell types. Embryonic cells from different developmental stages are included as controls. EPS-TPS-sub, TPS 2C-subpopulation from EPS-TPS cells; 2C-TPS-sub, TPS 2C-subpopulation from 2C-TPS cells; TBLC-sup, subpopulation from TBLCs; 2CLC, spontaneous 2C-like cells. **d** ssGSEA analysis showing the similarities between embryonic cells from different developmental stages and in vitro cell types. EPS-TPS-sub, TPS 2C-subpopulation from EPS-TPS cells; 2C-TPS-sub, TPS 2C-subpopulation from 2C-TPS cells; TBLC-sup, subpopulation from TBLCs; 2CLC, spontaneous 2C-like cells. **e** Comparison of regulon activities between TPS 2C-subpopulation (TPS-sub) and EPS cells. Regulons that are enriched in TPS 2C-subpopulation and EPS cells are highlighted. **f** Analysis of regulon activities at different developmental stages. The activities of top 20 regulons that are upregulated and downregulated in TPS 2C-subpopulation (TPS-sub) are quantified. **g** Identification of open and closed peaks that are enriched in TPS cells. ES and EPS cells were used for comparison. Orange color (high) indicates TPS-enriched open peaks, and blue color (low) indicates TPS-enriched closed peaks. **h** Average signal intensities of TPS-enriched open and closed peaks in TPS, ES, EPS cells and 2-cell embryos. Orange color indicates TPS-enriched open peaks, and blue color indicates TPS-enriched closed peaks. “ES: this study” indicates sequencing data of ES cell samples that were collected and sequenced in this study. “ES: public” indicates sequencing data of ES cells from public resources. **i** Comparison of global DNA CpG methylation levels in TPS, ES, EPS cells and embryonic cells from different developmental stages (2-cell, 4-cell, E6.5 and E7.5 embryos, E3.5 inner cell mass (ICM)).

To further explore the transcriptomic features of TPS cells, we performed single-cell RNA-seq using TPS cells. As controls, the single-cell RNA-seq data of mouse EPS cells, ES cells, 2C-like cells and recently reported totipotent blastomere-like cells (TBLCs) were also analyzed.^14,21^ Consistent with bulk RNA-seq analysis, the majority of TPS cells expressed multiple totipotency marker genes (Fig. 2a), and their expression levels varied among the population. Among the two TPS cell lines analyzed, the percentage of *Zscan4*/*MERVL* double positive cells are 16.70% (of 10,302 cells) and 14.75% (of 8364 cells), respectively. Notably, the varied expression of totipotency marker genes was also observed in TBLCs (Supplementary information, Fig. S5a), which is consistent with a recent report.^22^ We further compared the transcriptome of these in vitro cell types with those of early embryos from 2-cell to E7.5 stages (Fig. 2b), and applied an analytical technique based on quadratic programming to quantify the transcriptomic similarity of these cell types to 2-cell embryos.^23^ The identity score of TPS cells was higher than those of other cell types (Fig. 2c), suggesting that TPS cells transcriptomically more resemble 2-cell embryos compared to other cell types.

Because the expression of totipotency marker genes varied among the TPS cell population, we further explored the subpopulations that reside in the TPS cell population, and identified a portion of cells (on average 9.14%) in the TPS cell population that transcriptomically resembled middle-to-late 2-cell embryos (Fig. 2b). Importantly, the identity score of this TPS subpopulation (TPS 2C-subpopulation) is the highest among all the in vitro cell types and subpopulations analyzed (Fig. 2c), which was also supported by single sample gene set enrichment analysis (ssGSEA) (Fig. 2d). We also analyzed the transcriptomic difference between the TPS 2C-subpopulation and 2-cell embryos. GO analysis revealed that genes differently expressed between this TPS 2C-subpopulation and 2-cell embryos are majorly involved in the regulation of mitotic cell cycle, proteasomal protein catabolism, nucleoside triphosphate metabolism, and oxidative phosphorylation (Supplementary information, Table S2). Using Single-Cell rEgulatory Network Inference and Clustering (SCENIC), we further analyzed the regulatory network of the TPS 2C-subpopulation and compared it with that of EPS cells. Notably, the activities of totipotency regulators were upregulated in this population, whereas those of pluripotency regulators were downregulated (Fig. 2e, f). Collectively, these data suggest that TPS cells shared totipotent transcriptional features with 2-cell embryos and a TPS cell subpopulation transcriptomically resembled middle-to-late 2C embryos.

### TPS cells share epigenetic features with 2-cell blastomeres

To characterize the epigenetic features of TPS cells, we first performed ATAC-seq for transposase-accessible chromatin on these cells at the single-cell level. As controls, EPS cells and ES cells were also analyzed. Compared to EPS and ES cells, we identified 1857 open and 8903 closed peaks that were uniquely enriched at annotated or putative enhancers and promoters in TPS cells (Fig. 2g). We further analyzed the distribution of TPS-specific open and closed peaks in 2-cell embryos and mouse ES cells. Notably, TPS-specific open loci were also highly opened in 2-cell embryos, whereas TPS-specific closed loci were in a more closed state in 2-cell embryos (Fig. 2h). We further analyzed the global DNA methylation profiles of TPS, EPS and ES cells using whole-genome bisulfite sequencing (WGBS). The global DNA methylation level in TPS cells was greatly reduced compared to those in EPS and ES cells (Fig. 2i), but similar to that in 2-cell embryos. In addition, we also observed that the DNA methylation levels were reduced in the loci of representative totipotency genes, such as *Zfp352*, *Zscan4d*, and *Tcstv1* (Supplementary information, Fig. S5b). Taken together, these results suggest that TPS cells share epigenetic features with 2-cell blastomeres in the aspects of accessible chromatin landscape and DNA methylation profiles.

### TPS cells can generate both embryonic and extraembryonic lineages in vivo

To explore the developmental potentials of TPS cells in vivo, we first injected these cells into immune-deficient mice and obtained teratomas, and analyzed these teratomas using single-cell RNA-seq. Notably, in addition to the presence of multiple embryonic lineages in the teratomas, we found that teratomas derived from TPS cells also contained extraembryonic lineages (Supplementary information, Fig. S6a, b). Next, we examined the in vivo chimera-forming ability of TPS cells, and found that TPS cells after 5 passages showed relatively higher chimera-forming ability (Supplementary information, Table S1). We also tested the germline competence of TPS cells by injecting them into 8-cell embryos and transplanting the injected embryos in vivo to generate chimeric embryos. Analysis of the E13.5 chimeric embryos showed that derivatives of these cells can efficiently form chimeras in the fetal gonads, which expressed OCT4-EGFP (Supplementary information, Fig. S6c). In addition, chimeric mice were also generated (Supplementary information, Fig. S6c).

**Fig. 3:**
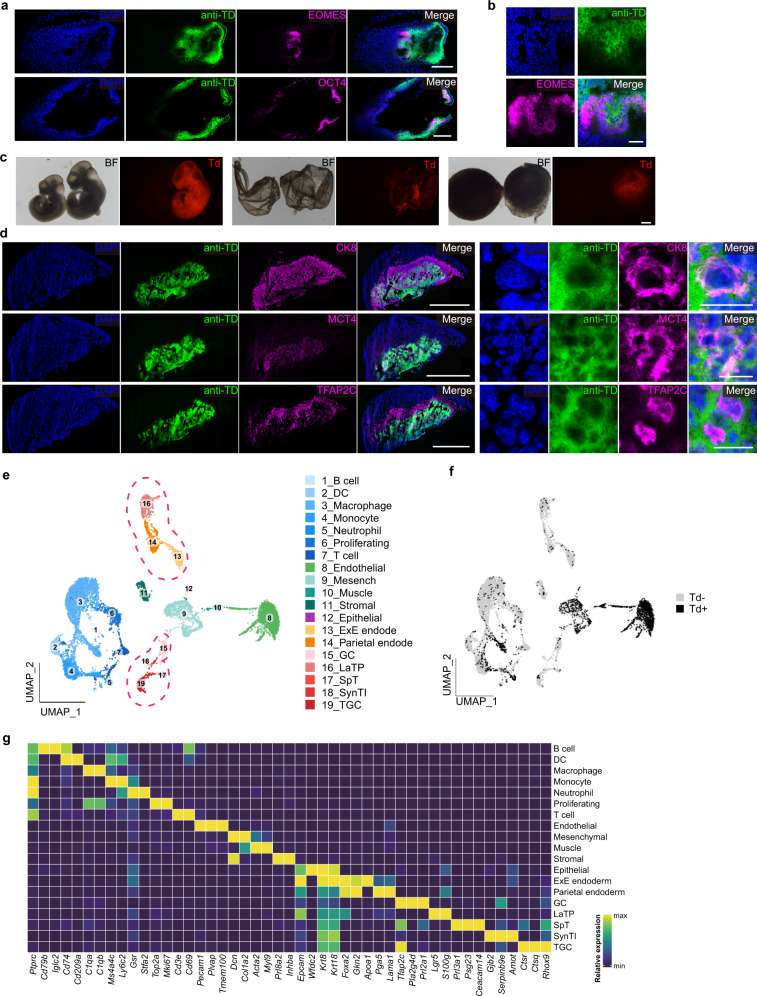
TPS cells can generate both embryonic and extraembryonic lineages in vivo. **a** Representative immunofluorescent analysis of expression of embryonic (OCT4) and extraembryonic ectoderm (EOMES) markers in chimeric derivative cells from single TPS cells in E7.5 conceptuses. The stained sections were from the same analyzed conceptus. Scale bars, 200 μm. anti-TD, immuno-staining of tdTomato protein. Similar images were obtained in at least 3 independent experiments. **b** Enlarged images showing contribution of single TPS derivatives in the extraembryonic ectoderm region. Scale bar, 50 μm. anti-TD, immuno-staining of tdTomato protein. **c** Representative images showing contribution of single TPS derivative cells in E10.5 embryo, yolk sac and placenta. Scale bar, 500 μm. Td, endogenous tdTomato. BF, bright field. Similar images were obtained in at least 3 independent experiments. **d** Representative immunofluorescent analysis of E10.5 chimeric placenta generated by injection of single TPS cells (tdTomato labeled). 2-cell embryo-derived TPS cells were used. The left panels show the original images and the right panels show the enlarged images. anti-TD, immuno-staining of tdTomato protein. Scale bars, 2 mm (left panels) and 20 μm (right panels). Similar images were obtained in at least 3 independent experiments. **e** UMAP plot showing the 19 main clusters in E17.5 chimeric placenta. Pink dotted line indicates extraembryonic cell lineages. **f** UMAP plot showing tdTomato expression in E17.5 chimeric placenta. Td+, tdTomato-positive cells. Td–, tdTomato-negative cells. **g** Heatmap showing the average expression of specific marker genes for each cluster from **f**.

To rigorously analyze the developmental potentials of TPS cells, we tested their ability of generating embryonic and extraembryonic lineages in E7.5 mouse embryos in vivo. To this end, TPS cells that continuously expressed tdTomato were injected into 8-cell embryos which were transferred in vivo. As a control, single 8-cell blastomeres were also injected. Notably, we observed the contribution of single TPS derivative cells to both embryonic and extraembryonic regions in E7.5 mouse chimeric embryos (Supplementary information, Table S1). Immunofluorescent analysis further confirmed that chimeric extraembryonic cells expressed EOMES in the region of extraembryonic ectoderm (ExE) (Fig. 3a, b), suggesting that they adopted an extraembryonic trophoblast fate. Meanwhile, we also noticed that cells that integrated into the visceral endoderm (VE) region expressed the VE markers SOX17 (Supplementary information, Fig. S7a), suggesting their VE identity.

Next, we analyzed the developmental potentials of single TPS cells in E10.5 mouse conceptuses (Supplementary information, Table S1). The injected mouse embryos were transferred in vivo and recovered at E10.5 stage. Notably, similar to blastomere of 8-cell embryos (Supplementary information, Fig. S7b), chimeric contribution of single TPS derivative cells to embryo, placenta and yolk sac was observed (Fig. 3c), which was also confirmed by FACS analysis (Supplementary information, Fig. S7c). To further confirm the trophoblast identity of chimeric cells in the placenta, we performed immunofluorescent analysis. Similar to the positive control of single 8-cell blastomere (Supplementary information, Fig. S7e), derivatives of TPS cells integrated into the trophoblast layers of the chimeric placentas and expressed the trophoblast markers CK8, MCT4 and TFAP2C (Fig. 3d; Supplementary information, Fig. S7d), suggesting that these chimeric cells can further differentiate into trophoblast lineages. As the negative control, we did not detect the expression of tdTomato in the non-injected placentas (Supplementary information, Fig. S7f).

To further analyze the trophoblast identity of chimeric cells in the placenta at late developmental stages, we generated E17.5 chimeric conceptuses using TPS cells (Supplementary information, Fig. S8a). We performed single-cell RNA-seq analysis using cells from the chimeric E17.5 placenta (Fig. 3e). Among the 9765 analyzed cells from the chimeric placenta, 1924 cells expressed tdTomato (Fig. 3f), indicating that they are derivatives of TPS cells. Notably, these TPS derivatives contained extraembryonic lineages expressing trophoblast subtype markers such as *Ctsq* (trophoblast giant cells), *Prl3a1* (spongiotrophoblasts), *Pla2g4d* (glycogen trophoblast cells), and *Lgr5* (labyrinth trophoblast progenitor) (Fig. 3g). Moreover, these TPS-derived trophoblast cells are transcriptomically similar to their in vivo counterparts (Fig. 3e, f). These results were further supported by qPCR analysis using FACS-purified chimeric TPS derivatives from E18.5 placenta (Supplementary information, Fig. S8b, c). To exclude the potential artifact of cell fusion in the placenta, we injected tdTomato reporter-labeled TPS cells into mouse 8-cell embryos that expressed EGFP. Importantly, the majority of chimeric tdTomato^+^ cells in the placenta did not show EGFP signal (Supplementary information, Fig. S8b). Collectively, the chimeric analyses provide strong evidence that TPS cells have extraembryonic and embryonic developmental potentials in vivo at the single-cell level.

### Induction of blastocyst-like structures from TPS cells in vitro

**Fig. 4:**
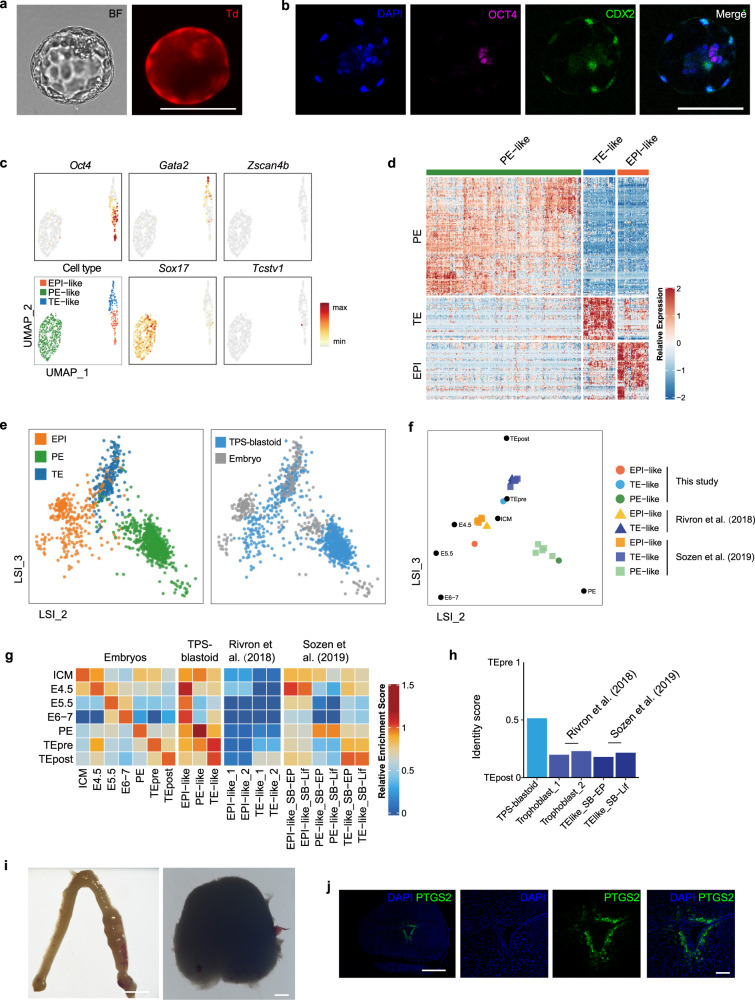
Induction of blastocyst-like structures from TPS cells in vitro. **a** Representative morphology of blastoids induced from TPS cells. Scale bar, 100 μm. BF, bright field. Td, endogenous tdTomato. Similar images were obtained in at least 3 independent experiments. **b** Representative immunofluorescent analysis of trophectoderm (CDX2) and epiblast (OCT4) marker genes in TPS-blastoids. Scale bars, 100 μm. Similar images were obtained in at least 3 independent experiments. **c** UMAP plot showing the expression of representative marker genes for epiblast, trophectoderm, primitive endoderm, and totipotency in TPS-blastoid cells at the single-cell level. EPI-like, epiblast-like cells; PE-like, primitive endoderm-like cells; TE-like, trophectoderm-like cells. **d** Heatmap showing expression of 262 representative marker genes for epiblast, primitive endoderm and trophectoderm in TPS-blastoid cells. EPI-like, epiblast-like cells; PE-like, primitive endoderm-like cells; TE-like, trophectoderm-like cells. **e** LSI analysis comparing cells from E4.5 blastocysts and TPS-blastoids. Left panel shows cell lineage assignments, and right panel shows plots for blastocyst and TPS-blastoids. EPI, epiblast; PE, primitive endoderm; TE, trophectoderm. **f** LSI analysis comparing embryonic cells from different developmental stages with blastoid cells from different studies. The single-cell RNA-seq data of blastoid cells from this study, Rivron et al.^28^ and Sozen et al.^29^ were analyzed. ICM, E3.5 inner cell mass; E4.5, E4.5 epiblast; E5.5, E5.5 epiblast; E6–7, E6–7 epiblast; PE, primitive endoderm (E4.5–E7.5); TEpre, trophectoderm from E3.5–E4.5 blastocysts; TEpost, E5.25–E6.5 extraembryonic ectoderm; EPI-like, epiblast-like cells; PE-like, primitive endoderm-like cells; TE-like, trophectoderm-like cells. **g** ssGSEA analysis showing the similarities between embryonic cells from different developmental stages and blastoid cells from different studies. The single-cell RNA-seq data of blastoid cells from this study, Rivron et al.^28^ and Sozen et al.^29^ were analyzed. SB-EP indicates cells from EPS/TS-blastoids. SB-Lif indicates cells from ES/TS-blastoids. **h** Quadratic programming-based deconvolution analysis showing the transcriptomic similarity between preimplantation trophectoderm and trophectoderm-like cells from different studies. **i** Representative images showing the formation of decidua in the mouse uterus 4 days after TPS-derived blastoids were transferred at 2.5 dpc. Scale bars, 5 mm (left image) and 500 μm (right image). Similar images were obtained in at least 3 independent experiments. **j** Representative immunofluorescent analysis of PTGS2 expression in TPS-derived decidua. Scale bars, 500 μm (left) and 100 μm (right). Similar images were obtained in at least 2 independent experiments.

Because TPS cells have embryonic and extraembryonic developmental potentials, we explored whether they can be induced into blastocyst-like structures (blastoids) in vitro. FGF, BMP and Yap signalings are important for preimplantation development especially for extraembryonic lineage development,^24–27^ and we attempted to treat TPS cells with bFGF, FGF4, BMP4 and LPA. TPS cells were seeded onto AggreWell microwell plate and treated with these factors, resulting in small aggregates. Notably, these aggregates could further form cavity and morphologically resemble preimplantation blastocysts (Fig. 4a; Supplementary information, Table S1). Immunofluorescent analysis showed that blastoids induced from TPS cells expressed trophectoderm (CDX2) and epiblast (OCT4) markers (Fig. 4b).

To analyze the transcriptome features of TPS-blastoids, we performed single-cell RNA-seq analysis. Cluster analysis divided the analyzed 914 cells into 3 clusters, and these 3 clusters expressed markers of epiblast, primitive endoderm and trophectoderm, respectively, including *Oct4* (epiblast), *Sox17* (primitive endoderm) and *Gata2* (trophectoderm) (Fig. 4c). Among 914 analyzed cells from the blastoids, 424 cells are *Cdx2* positive (46.4%) and 176 cells are *Oct4* positive (19.2%). It is also notable that the expression of totipotency marker genes was nearly absent in the 3 clusters (Fig. 4c), suggesting that cells in the TPS-blastoids lose totipotent features. Next, a panel of 262 representative lineages markers of epiblast, primitive endoderm and trophectoderm were analyzed in the TPS-blastoid cells. Consistent with cluster analysis, the three clusters clustered well into epiblast-, primitive endoderm- and trophectoderm-like lineage, respectively (Fig. 4d). We further compared the transcriptomic features of these TPS-blastoid cells with those of E4.5 blastocyst, and found that most of the TPS-blastoid cells clustered together with blastocyst cells assigned to the same lineage (Fig. 4e).

We further analyzed the transcriptomic similarity between TPS-blastoid cells and blastocyst cells. Epiblast cells (E4.5–E7.5) and extraembryonic ectoderm cells from the post-implantation stages (E5.25–E6.5) were also included in the analysis. As controls, we also analyzed blastoids generated by combining mouse TS cells with mouse ES or EPS cells.^28,29^ Importantly, cells resembling the epiblast, primitive endoderm and trophectoderm in the TPS-blastoids were transcriptionally similar to the three lineages from the E4.5 blastocyst stage (Fig. 4f). Notably, compared to trophectoderm-like cells from TPS-blastoids, there are significant transcriptomic differences between trophectoderm-like cells from previously reported blastoids and in vivo trophectoderm (Fig. 4f). These results were further supported by ssGSEA and quadratic programming-based identity score calculation (Fig. 4g, h). Collectively, these data suggest that TPS-blastoids contain the three lineages of blastocysts, transcriptomically resembling E4.5 blastocyst.

We further evaluated the in vivo developmental potentials of TPS-blastoids. To this end, tdTomato reporter-labeled TPS-blastoids were transferred into pseudopregnant mice at 2.5 days post coitum (dpc). At 6.5 dpc, decidualization was induced by TPS-blastoids (Fig. 4i, j), which was further evidenced by the induction of PTGS2 expression in the deciduae. These results suggest that TPS-blastoids can implant and trigger decidualization in vivo.

### Mechanistic exploration of totipotency induction and maintenance in TPS cells

**Fig. 5:**
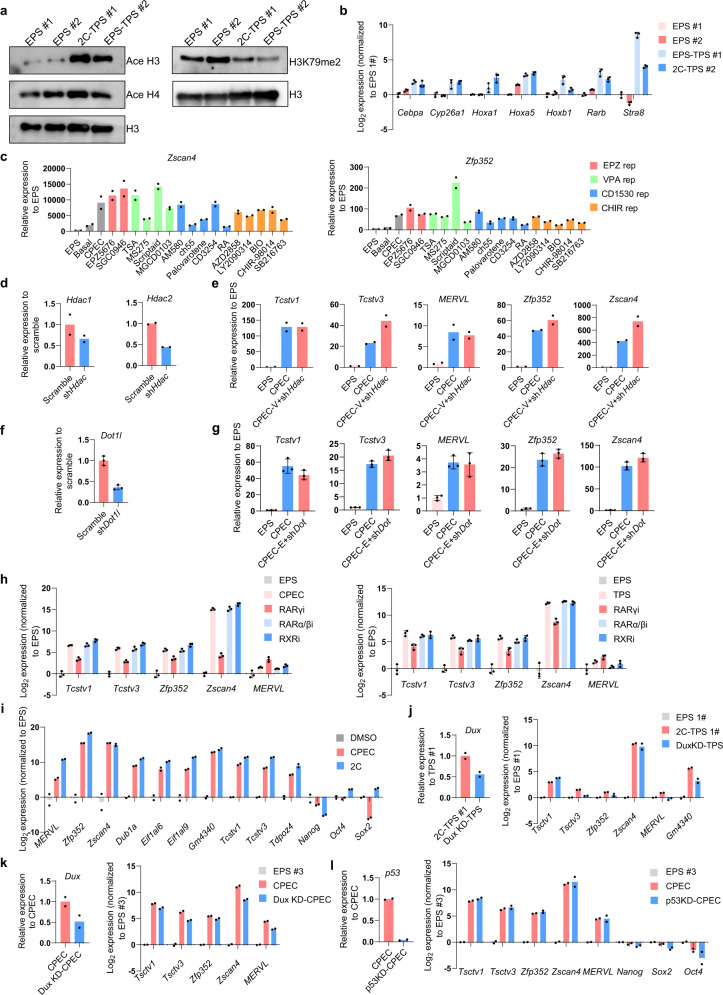
Mechanistic exploration of totipotency induction and maintenance in TPS cells. **a** Western blot analysis showing the levels of histone H3 and H4 acetylation and H3K79me2 of EPS and TPS cells. Similar results were obtained in at least 2 independent experiments. **b** qPCR analysis of expression levels of classical RAR downstream target genes in EPS and TPS cells. *n* = 3 biological replicates. **c** qPCR analysis of expression levels of representative totipotency marker genes on day 3 upon treatment of different small molecule combinations. In the CPEC condition, EPZ004777, VPA, CD1530 and CHIR 99021 were replaced by small molecules targeting DOT1L, HDAC, RA signaling and GSK3β, respectively. *n* = 2 technical replicates. Similar results were obtained in at least 2 independent experiments. EPS, EPS cells; Basal, EPS cells cultured in the basal medium of CPEC condition. EPZ rep, VPA rep, CD1530 rep and CHIR rep indicate small molecules that target DOT1L, HDAC, RA and GSK3β, respectively. **d** qPCR analysis of *Hdac1* and *Hdac2* expression in EPS cells after shRNA knockdown of *Hdac1/2*. sh*Hdac*, *Hdac1/2* shRNA. *n* = 2 biological replicates. **e** qPCR analysis of expression levels of representative totipotency marker genes on day 3 after knocking down *Hdac1/2* in EPS cells during TPS cell induction. EPS, EPS cells; CPEC-V + sh*Hdac*, replacement of VPA with *Hdac1/2* knockdown. *n* = 2 biological replicates. **f** qPCR analysis of *Dot1l* expression in EPS cells after shRNA knockdown of *Dot1l*. sh*Dot1l*, *Dot1l* shRNA. *n* = 3 biological replicates. **g** qPCR analysis of expression levels of representative totipotency marker genes on day 3 after knocking down *Dot1l* in EPS cells during TPS cell induction. EPS, EPS cells; CPEC-E + sh*Dot*, replacement of EPZ004777 with *Dot1l* knockdown. *n* = 3 biological replicates. **h** qPCR analysis of the effect of inhibiting RAR signaling on totipotency induction (left panel) and maintenance (right panel) in TPS cells. Expression of representative totipotency marker genes were analyzed. RARγi, LY2955303; RARα/βi, LE135; RXRi, UVI3003. *n* = 3 biological replicates. **i** qPCR analysis of the effect of CPEC chemical cocktail on totipotency maintenance in early preimplantation embryos. Small molecules were added from the 2-cell embryo stage for 2 days. DMSO was used as a negative control. 2C, 2-cell embryo. *n* = 2 biological replicates. **j** qPCR analysis of the effect of *Dux* knockdown on maintaining totipotency in TPS cells. Expression of representative totipotency marker genes were analyzed. Dux KD, *dux* knockdown. *n* = 2 biological replicates. **k** qPCR analysis of the effect of *Dux* knockdown on inducing totipotency from EPS cells. Expression of representative totipotency marker genes were analyzed. Dux KD, *dux* knockdown. *n* = 2 biological replicates. **l** qPCR analysis of the effect of *p53* knockdown on inducing totipotency from EPS cells. Expression of representative totipotency marker genes were analyzed. p53 KD, *p53* knockdown. *n* = 2 biological replicates.

We attempted to explore the mechanism of totipotency regulation by the CPEC condition. Because VPA, CD1530 and EPZ004777 showed synergistic effects on induction of totipotency marker gene expression, we first focused on analyzing the molecular targets of these three compounds. Among the 3 small molecules, VPA is reported to be an HDAC inhibitor, CD1530 an RARγ agonist, and EPZ004777 a Dot1L inhibitor. To test whether these small molecules indeed target these molecular targets in TPS cells, we first analyzed the levels of histone acetylation and H3K79 methylations in TPS cells by western blotting. Compared to that in EPS cells, we found that the histone H3 acetylation level was increased whereas the H3K79 methylation level was decreased (Fig. 5a). In addition, upregulation of classical RAR downstream target genes in TPS cells was observed (Fig. 5b). Interestingly, we found that the chromatin accessibility of RARγ-binding loci in TPS cells is reduced compared to that in EPS cells, and a similar result was observed in 2-cell embryos (Supplementary information, Fig. S9a).

To confirm that these small molecules indeed induce totipotency by targeting these reported targets, we first replaced VPA, EPZ004777 and CD1530 respectively with other small molecules targeting the same molecular targets, including HDAC, DOT1L, and RAR. We found that these replacements can also support the induction of totipotency marker gene expression (Fig. 5c; Supplementary information, Fig. S9b). Consistent with these results, knockdown of *Hdac1/2* and *Dot1l* could also replace VPA and EPZ004777 in induction of totipotency markers, respectively (Fig. 5d–g), and inhibition of RARγ signaling by small molecules greatly reduced the expression of totipotency markers during TPS induction and maintenance (Fig. 5h). Interestingly, qPCR analysis showed that the CPEC treatment of preimplantation embryos could facilitate the maintenance of totipotency marker gene expression in preimplantation mouse embryos beyond the 2-cell embryo stage (Fig. 5i). Taken together, these data suggest that inhibition of HDAC1/2 and DOT1L activity and activation of RARγ signaling are important for inducing totipotency in TPS cells.

Next, we sought to analyze the role of CHIR 99021 in the induction and maintenance of TPS cells. Consistent with its reported role of activating Wnt signaling by inhibiting GSK3β,^30^ TPS cells expressed β-catenin at the protein level (Supplementary information, Fig. S9c). To examine whether CHIR 99021 is required for maintaining TPS cells, we removed CHIR 99021 from the chemical cocktail during culturing of TPS cells. The omission of CHIR 99021 led to reduced proliferation during the first few passages (~3–5 passages). After further passaging, we found that CHIR 99021 is not required for the proliferation of TPS cells (data not shown). qPCR analysis further showed that the expression of totipotency marker genes in TPS cells is not affected by the omission of CHIR 99021 after long-term culturing (Supplementary information, Fig. S9d). However, we also found that the omission of CHIR 99021 can greatly reduce the in vivo chimera-forming potential of TPS cells (Supplementary information, Table S1). Therefore, CHIR 99021 is beneficial for promoting proliferation during TPS conversion and important for maintaining the in vivo developmental potency of TPS cells.

Finally, we attempted to explore the roles of known key totipotency and pluripotency regulators in the induction and maintenance of TPS cells. *Dux* is an important regulator in induction of 2C-like cells,^31,32^ and *p53* is reported to be a key upstream activator of *Dux* in induction of 2C-like cells.^33^ During the maintenance and induction of TPS cells, we found that knockdown of *Dux* expression led to downregulation of several totipotency marker genes (*MERVL*, *Zfp352*, *Tcstv3*), whereas expression of other totipotency marker genes (*Tcstv1*, *Zscan4*) was not significantly affected (Fig. 5j, k). On the other hand, *p53* knockdown did not cause decreased expression of totipotency marker genes during the induction of TPS cells (Fig. 5l). Next, we examined the role of *Oct4* and LIF signaling, the key pluripotency regulators,^34^ in the maintenance of TPS cells. Knockout of *Oct4* in TPS cells did not significantly affect the expression of totipotency marker genes (Supplementary information, Fig. S10a), whereas the proliferation of TPS cells was reduced (Supplementary information, Fig. S10b). Similar to *Oct4*, inhibition of LIF signaling by small molecules also led to an impaired proliferation of TPS cells (Supplementary information, Fig. S10c), and the expression of totipotency marker genes was not affected (Supplementary information, Fig. S10d). Collectively, these data suggest that the mechanism of totipotency regulation in TPS cells is unique and distinct from that in 2C-like cells, which requires further exploration.

## Discussion

In this study, we reported a chemical cocktail that can support the derivation of totipotent-like stem cells from 2-cell mouse embryos and EPS cells. TPS cells showed transcriptomic and epigenetic features that resemble 2-cell mouse embryos. Importantly, TPS cells have the capacity to generate both embryonic and extraembryonic lineages in vivo at the single-cell level. When self-organizing in vitro, TPS cells can form blastocyst-like structures, which transcriptomically resemble E4.5 blastocysts and can implant and trigger decidualization in vivo. Mechanistic studies showed that HDAC1/2 and DOT1L inhibition as well as RARγ signaling activation are important for inducing and maintaining totipotent features of TPS cells. These results demonstrate the feasibility of deriving and maintaining totipotent-like stem cells in vitro.

The derivation of TPS cells from 2-cell mouse embryos represents an important step toward capturing authentic totipotency in vitro. A long-standing scientific question in the stem cell field is whether totipotent stem cells can be captured from totipotent embryos,^13^ which has not been achieved. Efforts to generate totipotent-like cells, including 2C-like cells and TBLCs,^14,21^ rely solely on the conversion from pluripotent stem cells and have not been made on totipotent embryos. In contrast, our results showed that the treatment of 4 small molecules (CPEC) on preimplantation embryos could facilitate the maintenance of totipotency marker gene expression in preimplantation mouse embryos beyond the 2-cell embryo stage (Fig. 5i). Moreover, the CPEC condition permitted establishment of 2-cell embryo-derived TPS cells, which highly expressed totipotency marker genes (Fig. 1c), shared transcriptomic and epigenetic features with 2-cell embryos (Fig. 2), and have bi-directional developmental potentials which enables formation of blastoids (Figs. 3d, 4). These findings provide the proof-of-principle evidence that it is possible to directly derive totipotent-like stem cells from totipotent embryos, which represents an important step toward capturing bona fide 2-cell totipotent embryos in vitro.

Another key unique feature of TPS cells is their ability to self-organize to generate blastocyst-like structures, mimicking the natural developmental process. Upon the stimulation of natural signaling molecules that drive early preimplantation development, TPS cells can self-organize into blastoids which transcriptomically resemble in vivo E4.5 blastocyst (Fig. 4a–g). Moreover, these blastoids can implant and induce decidualization in vivo (Fig. 4i, j). Previous efforts on generation of synthetic blastocysts using pluripotent cells rely on the combined use of TS cells or reprogramming from epiblast stem cells.^28,35^ The major limitation of using TS cells is that TS-derived blastoids transcriptionally resemble postimplantation extraembryonic ectoderm (ExE) but not preimplantation trophectoderm,^36–38^ therefore the induction process is artificial and unnatural, leading to the significant transcriptomic differences between in vivo trophectoderm and trophectoderm-like cells from the TS-derived blastoids (Fig. 4f–h). Likewise, induction of blastocyst-like cysts (iBLCs) from epiblast stem cells by reprogramming does not mimic the natural developmental process.^35^ In contrast, the generation of TPS-blastoids relied on the differentiation of totipotent-like stem cells to the blastocyst lineages (Fig. 4c), which more closely resembles the process of blastocyst formation in vivo. On the other hand, unlike EPS-blastoids that contain a significant portion of intermediates and mesodermal cells,^18^ the majority of TPS-blastoids contained the three blastocyst lineages and intermediates were nearly absent (Fig. 4c, d). Therefore, TPS-blastoids avoid the limitations of previously reported synthetic blastocysts and could serve as a powerful model to recapitulate the in vivo process of blastocyst formation in vitro. It is also important to further optimize the TPS-based blastoid induction approach and explore whether they can develop into conceptuses in vivo.

Importantly, TPS cells provide a valuable in vitro platform to explore the regulation of totipotency, and clarifying the molecular targets of the small molecules used in the CPEC condition could provide a novel mechanistic insight into the regulation of totipotency.^13^ Importantly, we found that inhibition of HDAC1/2 and DOT1L activities and activation of RARγ signaling are important for inducing totipotency in TPS cells (Fig. 5a–h). Interestingly, the role of RARγ signaling in totipotency regulation is supported by a recent study showing that RA signaling is critical during the totipotency window in early development.^39^ It would be important to further explore the synergetic effect of these targets on maintenance of totipotency in embryos. Recent studies suggest that 2C-like cells do not fully recapitulate 2-cell embryos in terms of regulation of 2-cell embryo-specific genes and cautions should be taken when studying totipotency using 2C-like cells as the model system,^40^ highlighting the demand for establishing new in vitro model systems to study totipotency. Although one recent study showed the induction of TBLCs by spliceosome inhibition,^21^ this approach only enabled their conversion from pluripotent stem cells but not capturing from totipotent embryos, whereas our CPEC condition permits derivation of TPS cells from 2C embryos. In addition, the transcriptomic profiles of the TPS 2C-subpopulation are significantly closer to that of middle-to-late 2C embryos compared to that of TBLCs (Fig. 2b–d). Moreover, our CPEC condition also bypasses potential safety concerns associated with spliceosome inhibition, such as tumor induction or proliferation difficulty.^41–44^ Therefore, TPS cells have wide application potentials as a new platform favorable for studying totipotency in vitro.

While our study was being submitted for publication, a study related to this work was published,^45^ which reported an alternative chemical cocktail to support the generation of totipotent-like stem cells (TLSCs). Compared to our CPEC condition, the chemical cocktail reported by Yang et al.^45^ for generating TLSCs also included a DOT1L inhibitor, which further supports our finding that DOT1L inhibition is critical for totipotency regulation. Their chemical cocktail also contained other small molecules, such as A366 and AS8351. Nevertheless, targets of these small molecules and mechanistic roles in totipotency regulation were not studied. Most importantly, there are major differences between the study by Yang et al. and our work. First, Yang et al. developed a relatively complicated protocol to derive TLSCs from mouse preimplantation embryos. It is unclear whether the developmental potentials of these embryo-derived TLSCs were rigorously evaluated at the single-cell level or using the blastoid induction assay. In contrast, our CPEC condition supports derivation of TPS cells from both pluripotent cell lines and 2-cell embryos, and their developmental potentials have been extensively characterized (Figs. 3, 4; Supplementary information, Figs. S7 and S8). Second, unlike our study (Fig. 5i), the work by Yang et al. lacks important information of how their chemical cocktail impacted on totipotency marker genes in embryos beyond the 2-cell embryo stage, and it is unclear whether the process of generating TLSCs from the embryos reflected the nature of capturing totipotent cells, or inducing totipotency from embryonic cells. Third, despite the finding that blastoids were generated from the TLSCs, the transcriptomic similarity between TLSC-blastoids and in vivo blastocysts remains unknown. As a comparison, we performed single-cell RNA-seq analysis showing that TPS-blastoids transcriptomically resembled preimplantation mouse blastocysts. This study, together with our study, highlights the importance and utility of using the chemical approach to establish totipotent stem cells.

In summary, our study demonstrates the feasibility of deriving totipotent-like stem cells from early embryos. TPS cells would provide a useful tool for studying the mechanism of totipotency regulation as well as early preimplantation embryogenesis. Our study also opens up a new path toward capturing totipotent stem cells from other mammalian species including humans.

## Materials and methods

### Mice

The mouse strain B6-Tg (C57BL/6-tdTomato) was bought from Beijing Vitalstar Biotechnology Co., Ltd. Other mouse strains ICR and EGFP were purchased from Peking University Health Science Center Department of Laboratory Animal Science. All animal experiments were performed in accordance with the NIH guidelines. All mouse experiments were approved by the Institutional Animal Care and Use Committee of Peking University. The mice were housed in a temperature controlled room (22 ± 1 °C) with 40%–60% humidity, under a 12-h light/dark cycle between 06:00 and 18:00.

### Culture of mouse TPS cells

All cell lines were cultured under 20% O_2_ and 5% CO_2_ at 37 °C. Mouse TPS cells were cultured in CPEC medium containing N2B27 basal medium (50% DMEM/F12 (Gibco, 11330-032), 50% Neurobasal (Gibco, 21103-049), 0.5% N2 supplement (Gibco, 17502-048), 1% B27 supplement (Gibco, 12587-010), 1% GlutaMAX (Gibco, 35050-061), 1% nonessential amino acids (Gibco, 11140-050)) which promoted cell proliferation, or 1640 basal medium (RPMI Medium 1640 basic (Gibco, 22400-089), 0.5% N2 supplement (Gibco, 17502-048), 1% B27 supplement (Gibco, 12587-010), 1% GlutaMAX (Gibco, 35050-061), 1% nonessential amino acids (Gibco, 11140-050)) which enhanced totipotent molecular features, supplemented with 5% knockout serum replacement (Gibco, 10828-028), 0.2% sodium pyruvate (Gibco, 11360-070), 0.14% sodium DL-lactate solution (Sigma, L7900) and 0.1% Chemically Defined Lipid Concentrate (Gibco, 11905-031), VPA (50 μM; Selleck, S3944), CHIR-99021 (3 μM; Selleck, S1263), EPZ004777 (1 μM; Selleck, S7353) and CD1530 (0.2 μM–0.5 μM; Tocris, 2554). Mouse TPS cells were cultured on mitomycin C inactivated mouse embryonic fibroblast cells (feeders, 3 × 10^4^ cells/cm^2^). The culture medium was changed every day. TPS cells were passaged every three days with 0.05% trypsin- EDTA (Gibco, 25300-062) and seeded at a split ratio ranging from 1:3 to 1:10. To generate TPS cells, EPS cells or ES cells were dissociated and cultured on feeders in CPEC medium for the first three passages; EPS cells or ES cells were seeded at a lower split ratio of 1:3 to improve cell viability. After five passages, TPS cells were generated for further experiments.

### Derivation of mouse TPS cells from 2-cell embryos

TPS cells were derived from 2-cell embryos of B6-Tg mice. For the 2-cell embryos, the zona pellucida was removed by Acidic Tyrode’s Solution (Sigma, T1788). When the zona pellucida disappeared, the 2-cell embryos were washed three times by M2 medium (Sigma, M7167), and then, they were seeded on feeders in CPEC medium. 2–3 days later, the culture medium was replaced with the fresh CPEC medium. After 6 days, outgrowths were picked and dissected into small clumps. Four days later, outgrowths were picked again and digested by 0.05% trypsin-EDTA. Single cells were seeded on feeder in CPEC medium supplemented with Y27632 (10 μM; Tocris, 1254). TPS colonies emerged gradually. During the first five passages, it is recommended to seed the cells in CPEC medium supplemented with Y27632. On the second day, Y27632 was removed. The newly established cell lines were passaged using 0.05% trypsin-EDTA every three days, and used for further analysis.

### Transient overexpression of exogenous *Dux* in Zscan4-Emerald GFP and MERVL-tdTomato reporter cells

The plasmid containing exogenous *Dux* driven by CMV promoter was transfected into Zscan4-Emerald GFP and MERVL-tdTomato reporter cells by nucleofection. LCDM medium was used for culturing the cells after the transfection. Cells were harvested for further analysis after 3 days of transfection.

### Flow cytometry

Cells were gently digested into single cells using 0.05% trypsin-EDTA. Suspensions were filtered through a 40 μm cell strainer. Then, the samples were analyzed on CytoFLEX (Beckman Coulter). Data analysis was performed using CytExpert software.

### Cell sorting and culture of sorted cells

Cell sorting was performed on BD FACSAria SORP. MERVL-tdTomato-positive or Zscan4-Emerald GFP-positive cells were sorted and seeded on feeder with TPS medium or basal medium at density of 2 × 10^4^ cells/cm^2^. The culture medium was changed every day. The sorted cells were initially flat. However, the cells became domed after further passaging. Sorted cells were passaged every three days with 0.05% trypsin-EDTA (Gibco, 25300-062) and seeded at a split ratio ranging from 1:3 to 1:10.

### Karyotype analysis

Cells were prepared to give a 50%–70% confluence on day of sampling. After 2 h incubation with fresh medium, a Colcemid solution was added to the medium at a final concentration of 0.02 mg/mL and cells were incubated for 1 h. Then the cells were washed in PBS, trypsinized and spun down. To obtain a single cell suspension, the pellet was re-suspended in hypotonic solution (0.56% KCl), and left at room temperature for 6 min. After spinning and removing hypotonic solution, 5 mL of ice-cold fixative (3:1 methanol: acetic acid) was added dropwise to the suspension, which was left at room temperature for 5 min and then spun down. The fixing procedure was repeated for additional three times. Finally, the pellet was resuspended in a final volume of 1 mL fixative. The cells were then dropped onto slides washed by 5% acetic acid with or without ethanol (ice-cold) and stained with Giemsa. For each analysis, at least 30–40 metaphases were examined. The number of chromosomes as well as the presence of structural chromosomal abnormalities were examined.

### qPCR analysis

The total RNAs were isolated using the Direct-zol RNA Kits (ZYMO Research, R2052). RNA was converted to cDNA using TransScript First-Strand cDNA Synthesis SuperMix (TransGen Biotech, AT311). qPCR analysis was conducted using the KAPA SYBR FAST qPCR Kit (KAPA Biosystems, KK4601) with the Bio-Rad CFX Connect Real-Time System. The primers used for qPCR analysis are listed in Supplementary information, Table S3. The data were analyzed using the ΔΔCt method.

### qPCR analysis of telomere length in TPS cells

Genomic DNA was extracted from 2 × 10^5^ EPS cells, ES cells and TPS cells using the DNeasy Blood and Tissue Kit (Qiagen, 69506). Average telomere length was measured for total genomic DNA by qPCR as previously described.^46^ qPCR analysis was performed using the KAPA SYBR FAST qPCR Kit on Bio-Rad CFX Connect Real-Time System. The telomeric primers and 36B4 primers are listed in Supplementary information, Table S3. A sample of genomic DNA was diluted from 0.75 μg to 48 ng per well for standard curve calculation. The T/S ratio indicative of relative telomere length was calculated.

### In vitro induction of trophoblast stem-like cells from TPS cells

TPS cells were digested into single cells and seeded onto feeder cells, and cultured in TS conditioned medium: RPMI-1640 (Gibco, 11879-020) supplemented with 20% ES-qualified FBS, 1% L-glutamine (Gibco, 25030-081), 1% sodium pyruvate (Gibco, 11360-070), heparin (1 μg/mL; Macklin, H811552-500KU), mouse FGF4 (25 ng/mL; Bioteche, 5846-F4), human bFGF (20 ng/mL; Novoprotein, C046). After 3–4 days, cells were passaged using TS medium. Flat TS-like colonies gradually emerged after passaging. Then the culture medium was changed into serum-free FAXY-TS medium: 50% Neurobasal, 50% DMEM/F12, 0.5% N2 supplement, 1% B27 supplement, 1% L-glutamine, 1-thioglycerol (0.15 μM; Sigma, M6145), human bFGF (25 ng/mL; Novoprotein, C046), recombinant human activin A (20 ng/mL; Novoprotein, C687), XAV939 (10 μM; Selleck, S1180), and Y27632 (5 μM; Tocris, 1254).

### In vitro induction of primitive endoderm-like cells from TPS cells

TPS cells were differentiated to primitive endoderm-like cells over the course of 3 days by plating 2.5 × 10^4^ cells/cm^2^ onto matrigel-coated plates in N2B27 medium supplemented with mouse FGF4 (50 ng/mL; Bioteche, 5846-F4), retinoic acid (10 nM; Sigma, R2625), 8-Bromo cAMP (1 mM; Selleck, S7857), CHIR-99021 (3 μM; Selleck, S1263). After the emergence of XEN-like cells, XEN cells were maintained in eXEN medium: RPMI-1640 supplemented with 20% ES-qualified FBS, 1% sodium pyruvate, 1% L-glutamine, mouse FGF4 (25 ng/mL; Bioteche, 5846-F4) and heparin (1 μg/mL; Macklin, H811552-500KU).

### Immunofluorescence

The cells were fixed in 4% paraformaldehyde (DingGuo, AR-0211) at room temperature for 20 min and blocked with 3% normal donkey serum (Jackson ImmunoResearch, 017-000-121) in PBS (Corning, 21-040-CVR) plus 0.2% Triton X-100 (Sigma-Aldrich, T8787) at room temperature for 1 h. The cells were incubated with primary antibodies at 4 °C overnight. Then the cells were incubated with secondary antibodies (Jackson ImmunoResearch) at room temperature for 1 h after being washed 3 times with PBS. The nuclei were stained with DAPI (Roche Life Science, 10236276001) at room temperature for 3 min and washed 3 times with PBS. The following primary antibodies were used: anti-ZSCAN4 (1:5000; Millipore Sigma, AB4340), anti-MERVL-Gag (1:500; Epigentek, A-2801-100), anti-OCT4 (1:500; Abcam, ab181557), anti-OCT4 (1:200; Abcam, ab27985), anti-SOX2 (1:200; Millipore Sigma, AB5603), anti-EOMES (1:500; Abcam, ab183991), anti-TFAP2C (1:500; Abcam, ab218107), anti-PDGFRA (1:200; R&D, AF1062), anti-SOX17 (1:200; R&D, AF1924), anti-SOX7 (1:200; R&D, AF2766), anti-GATA6 (1:200; R&D, AF1700), anti-CDX2 (1:200; BioGenex, MU392A).

### Teratoma assay

Mouse TPS cells were collected by trypsinization before injection. Approximately 10^6^ cells were injected subcutaneously into immunodeficient NPG mice by mixing with Matrigel. Teratomas generally developed within 2–6 weeks, and the animals were killed before the tumor size exceeded 1.5 cm in diameter. The teratomas were then digested into single cells using Collagenase IV. Then red blood cells were removed using Red Blood Cell Lysis Buffer (Thermo Fisher Scientific, 00-4333-57). The digested teratoma cells were processed for 10× Genomics single-cell RNA-seq.

### Chimera formation assay by single-cell microinjection

Cells were digested by 0.05% trypsin-EDTA, and the digested cells were filtered through a 40 μm cell strainer and centrifuged at 1200–1500 rpm for 3 min at room temperature. The supernatant was removed, and the cells were resuspended using culture medium with the addition of Y-27632 (10 μM; Tocris, 1254) and placed on the ice before injection. After being placed on ice, the digested cells should be injected after 1 h; otherwise, another batch of cells were digested for the remaining injections.

Single cells were microinjected into 8-cell ICR diploid mouse embryos. The injected embryos were cultured in the culture medium with Y-27632 (10 μM; Tocris, 1254) for the first 4 h. For the generation of chimeric blastocysts, the embryos were transferred into the KSOM medium (Merck, MR-106-D). For the generation of single-cell-derived in vivo chimeric conceptuses, chimeric embryos were cultured in KSOM medium with Y-27632 (10 μM; Tocris, 1254) addition in a humidified incubator under 5% CO_2_ at 37 °C overnight. Injected embryos were transferred to uterine horns of 0.5 dpc pseudo-pregnant females. Fetal tissues, yolk sacs and placentas were dissected from conceptuses at E7.5, E10.5, E13.5 or E17.5 developmental stages.

### Chimera formation assay by multiple-cell microinjection

For the generation of chimeric blastocyst, TPS cells were cultured in FAXY basal medium (50% Neurobasal, 50% DMEM/F12, 0.5% N2 supplement, 1% B27 supplement, 1% L-glutamine, 1-thioglycerol (0.15 μM; Sigma, M6145), supplemented with human BMP4 (100 ng/mL; Stemimmune, HST-B4-0100) and bFGF (100 ng/mL; Novoprotein, C046)) for 2 days. The cells were first digested by 0.05% trypsin-EDTA, and filtered through a 40 μm strainer. Afterward, the cells were centrifuged at 1200–1500 rpm for 3 min, and then resuspended using culture medium and placed on ice. 10–15 of the digested cells were microinjected into each 2/8-cell ICR or GFP diploid mouse embryo. Injected embryos were transferred into KSOM medium and cultured in a humidified incubator under 5% CO_2_ at 37 °C for 48–72 h till developing to E4.5. The embryos were fixed and subjected to immunostaining, and > 20 embryos were analyzed.

### Chimera formation assay of single blastomere of 8-cell embryo

Immunofluorescent 8-cell embryos’ zona pellucida was removed by Acidic Tyrode’s Solution (Sigma, T1788). When the zona pellucida disappeared, the 2-cell embryos were washed 3 times with M2 medium (Sigma, M7167). The embryos were digested by TrypLE^TM^ Select (Gibco, A1217702) 3 times and then by DNase I (Gibco, 90083) twice. Blastomeres would separate after final digestion by TrypLE^TM^ Select and DNase I at 37 °C for 5 min. Single immunofluorescent blastomere was aggregated with a zona pellucida-removed wild-type 8-cell embryo in AggreWell (Stemcell, 34415). Aggregated embryos were cultured in KSOM in a humidified incubator under 5% CO_2_ at 37 °C overnight. Aggregated embryos were transferred to uterine horns of 2.5 dpc pseudo-pregnant females. Fetal tissues, yolk sacs and placentas were dissected from conceptuses at E7.5 or E10.5 developmental stages.

### Analysis of the contribution of TPS derivatives to E13.5 fetal gonads

TPS cells were microinjected into 8-cell ICR diploid mouse embryos. Injected embryos were transferred to uterine horns of 0.5 dpc pseudo-pregnant females. E13.5 embryos were collected for analyzing the expression of tdTomato and OCT4-GFP.

### Immunofluorescence of E4.5 embryo

E4.5 embryos were washed 3 times in PBS droplets, and then fixed in 4% paraformaldehyde (DingGuo, AR-0211) droplets at room temperature for 20 min. 3% normal donkey serum (Jackson ImmunoResearch, 017-000-121) in PBS (Corning, 21-040-CVR) plus 0.2% Triton X-100 (Sigma-Aldrich, T8787) were used to block embryos at room temperature for 1 h. Primary antibodies were diluted with blocking solution, and then incubated embryos at 4 °C overnight. The embryos were washed 3 times in PBS droplets, and then incubated with secondary antibodies (Jackson ImmunoResearch) at room temperature for 1 h. After final washes, blastocysts were transferred to confocal dish in PBS droplets covered with paraffin for imaging. Confocal microscopy imaging was performed using Leica TCS-SP8. The following primary antibodies were used: anti-tdTomato (1:2000; SICGEN, AB8181-200), anti-OCT4 (1:200; Santa Cruz Biotechnology, sc-8626), anti-PDGFRA (1:200; R&D, AF1062), anti-CDX2 (1:200; BIOGENEX, MU392A), anti-EOMES (1:500; Abcam, ab183991), anti-TFAP2C (1:500; Abcam, ab218107), anti-CK8 (1:200; Abcam, ab53280).

### Immunofluorescence of E7.5 embryo

E7.5 embryos were dissected in PBS, and then embedded in O.C.T (SAKURA, 4583). The embryos were frozen with liquid nitrogen vapor. Tissue sections of 10–15 mm thickness were cut in a cryostat microtome and transferred onto slides. The tissues were circled by PAP pen, then fixed in 4% paraformaldehyde (DingGuo, AR-0211) at room temperature for 15 min and blocked with 3% normal donkey serum (Jackson ImmunoResearch, 017-000-121) in PBS (Corning, 21-040-CVR) plus 0.2% Triton X-100 (Sigma-Aldrich, T8787) at room temperature for 1 h. After blocking, placental tissues were incubated with primary antibodies at 4 °C overnight. Placental tissues were incubated with secondary antibodies (Jackson ImmunoResearch) at room temperature for 1 h after being washed 3 times with PBS. The nuclei were stained with DAPI (Roche Life Science, 10236276001) at room temperature for 3 min and washed 3 times with PBS. Confocal microscopy imaging was performed using Leica TCS-SP8 and Leica TCS-SP8 DIVE. The following primary antibodies were used: anti-tdTomato (1:2000; SICGEN, AB8181-200), anti-OCT4 (1:500; Abcam, ab181557), anti-EOMES (1:500; Abcam, ab183991), anti-SOX17 (1:200; R&D, AF1924).

### Immunofluorescence of E10.5 placenta

E10.5 chimeric embryos were dissected in PBS. The fetus, yolk sac and placenta were separated using fine-pointed forceps. Placentas were embedded in O.C.T (SAKURA, 4583) and frozen with liquid nitrogen vapor. The tissues were circled by PAP pen, then fixed in 4% paraformaldehyde (DingGuo, AR-0211) at room temperature for 15 min and blocked with 3% normal donkey serum (Jackson ImmunoResearch, 017-000-121) in PBS (Corning, 21-040-CVR) plus 0.2% Triton X-100 (Sigma-Aldrich, T8787) at room temperature for 1 h. After blocking, placental tissues were incubated with primary antibodies at 4 °C overnight. Placental tissues were incubated with secondary antibodies (Jackson ImmunoResearch) at room temperature for 1 h after being washed 3 times with PBS. The nuclei were stained with DAPI (Roche Life Science, 10236276001) at room temperature for 3 min and washed 3 times with PBS. Confocal microscopy imaging was performed using Leica TCS-SP8 and Leica TCS-SP8 DIVE. The following primary antibodies were used: anti-tdTomato (1:2000; SICGEN, AB8181-200), anti-TFAP2C (1:500; Abcam, ab218107), anti-CK8 (1:500; Abcam, ab53280), anti-MCT4 (1:200; Millipore, AB3314P).

### Flow cytometry analysis of chimeric placental tissues

Chimeric EGFP^+^ placental tissues were gently isolated and digested into single cells using 0.1% Collagenase IV (Gibco, 17104019; dissolved in Ca^2+^/Mg^2+^-free PBS with 10% FBS and 100 μg/mL DNase I). Suspensions were filtered through a cell strainer (100 mm). Then, the samples were analyzed on an Arial Sorp (BD Biosciences) or CytoFLEX (Beckman Coulter) to detect the expression of tdTomato and EGFP. Data analysis was performed using FlowJo software (Ashland).

### Generation of blastoids

TPS cells were dissociated into single cells by 0.05% trypsin-EDTA. The cells were suspended in induction medium comprising: 1:1:1 mixture of TS conditioned medium, N2B27 basal medium and KSOM plus human BMP4 (100 ng/mL; Stemimmune, HST-B4-0100), human bFGF (100 ng/mL; Novoprotein, C046), mouse FGF4 (25 ng/mL; Bioteche, 5846-F4), LPA (10 μM; Sigma, 857228P) and Y27632 (10 μM; Tocris, 1254) on AggreWell (Stemcell, 34415). 3 × 10^4^ cells were seeded into one well. 4–6 days later, blastoids were formed.

### Immunofluorescence of blastoids

Blastoids were washed 3 times in PBS droplets, and then fixed in 4% paraformaldehyde (DingGuo, AR-0211) droplets at room temperature for 20 min. 3% normal donkey serum (Jackson ImmunoResearch, 017-000-121) in PBS (Corning, 21-485 040-CVR) plus 0.2% Triton X-100 (Sigma-Aldrich, T8787) were used to block embryos at room temperature for 1 h. Primary antibodies were diluted with blocking solution, and then incubated embryos at 4 °C overnight. The embryos were washed 3 times in PBS droplets, and then incubated with secondary antibodies (Jackson ImmunoResearch) at room temperature for 1 h. After final washes, blastoids were transferred to confocal dish in PBS droplets covered with paraffin for imaging. Confocal microscopy imaging was performed using Leica TCS-SP8. The following primary antibodies were used: anti-OCT4 (1:500; Abcam, ab181557), anti-CDX2 (1:200; BioGenex, MU392A).

### Western blot

For detection of histone proteins, the histone proteins were isolated from 5 × 10^6^ cells using histone protein extraction kit (beibokit, BB-3117). For detection of β-catenin protein, whole-cell protein extracts were isolated from 5 × 10^6^ cells using RIPA lysis buffer (Beyotime Technology Technology, P0013B) supplemented with protease inhibitor cocktail (Thermo Fisher Scientific, 78443) and phosphatase inhibitor cocktail (Thermo Fisher Scientific, 78428). The protein amount was determined using the Bicinchoninic acid (BCA) assay kit (Applygen, P1511). Blots were incubated in 5% skimmed milk powder/TBST at room temperature for 1 h, and then they were incubated with the following antibodies in 5% BSA or 5% skimmed milk powder/TBST at 4 °C overnight: anti-histone H3 (1:1000; Abcam, ab1791), anti-acetyl-histone H3 (1:1000; Millipore, 06-599), anti-acetyl-histone H4 (1:1000; Millipore, 06-866), anti-dimethyl-histone H3 (Lys79) (1:1000; Millipore, 04-835), anti-β-catenin (1:2000; Cell Signaling Technology, 8480), anti-GAPDH (1:4000; Applygen, C1312). The blots were washed using TBST, and further incubated with secondary antibodies for 1 h at room temperature while shaking. The following secondary antibodies were used: goat anti-rabbit IgG, HRP-linked antibody (1:3000; ZSGB-BIO, ZB-2301) and goat anti-mouse IgG, HRP-linked antibody (1:3000; ZSGB-BIO, ZB-2305). The blots were developed using western blotting luminol reagent (Santa Cruz, sc-2048).

### Generation of *p53* and *Dux* knockdown mEPS and mTPS cell lines

mEPS or mTPS cells carrying a Zscan4-Emerald GFP reporter were co-transfected with px330 plasmids (Addgene, 42230) encoding Cas9 and sgRNAs by nucleofection (4D-Nucleofector System, Lonza). The whole cells were collected to extract total RNAs for further analysis by qPCR.

### shRNA knockdown

*Hdac1/2* or *Dot1l* shRNAs were transfected into EPS cells by nucleofection (4D-Nucleofector System, Lonza). Then EPS cells were cultured in LCDM for two days. On the third day, EPS cells were transfected with the same shRNAs by nucleofection, and the cells were seeded in the CPEC medium or CPEC medium without VPA/EPZ004777. The medium was changed daily. Puromycin (0.8 μg/mL) was added in the culture medium to enrich cells expressing shRNAs. After three days, the cells were collected and total RNAs were isolated for qPCR analysis.

### Induction of *Oct4* knockout in TPS cells

*Oct4* conditional knockout mouse ES cell line ZHBtc4^47^ were converted to TPS cells using the CPEC condition for > 5 passages. Then tetracycline (1 μg/mL) was added in the CPEC medium to induce *Oct4* knockout in ZHBtc4-TPS cells. After 3 days of treatment, the proliferation state of these cells was examined. Then treated and untreated cells were collected and total RNAs were isolated for qPCR analysis.

### Analysis of the effect of CPEC chemical cocktail on totipotency maintenance in mouse preimplantation embryos

Mouse middle 2-cell embryos were collected and treated with the CPEC chemical cocktail (500 μM VPA, 1 μM EPZ004777, 0.5 μM CD1530, 3 μM CHIR 99021) in the KSOM medium. The KSOM medium supplemented with DMSO was used as a control. 48 h later, embryos were collected and lysed using Trizol reagent. Total RNAs were extracted from the lysates, and further used for cDNA synthesis. cDNAs were prepared and amplified using the Smart-seq2 approach. The amplified cDNA product was diluted 10-fold as required by the qPCR template. qPCR analysis was conducted using the KAPA SYBR FAST qPCR Kit (KAPA Biosystems, KK4601) on a Bio-Rad CFX Connect Real-Time System. The primers used for qPCR are listed in Supplementary information, Table S1.

### Analysis of the effects of HDAC inhibition, DOT1L inhibition, RAR activation/inhibition and LIF signaling on induction of totipotency in TPS cells using small molecules

EPS cells were seeded onto feeder cells. VPA, EPZ004777, CD1530 and CHIR 99021 were removed from the CPEC medium individually, and small molecules target the same target were added, respectively. After 3 days of induction, the cells were collected for further analysis. Small molecules replacing VPA included TSA (1 nM; Selleck, S1045), MS275 (1 μM; Selleck, S1053), Scriptaid (1 μM; Selleck, S8043), MGCD0103 (20 nM; Selleck, S1122). Small molecules replacing EPZ004777 included EPZ5676 (3 μM; Selleck, S5676) and SGC0946 (0.05 μM; Selleck, S7079). Small molecules replacing CD1530 included AM580 (0.05 μM; Selleck, S2933), ch55 (0.05 μM; MCE, HY-107397), Palovarotene (0.01 μM; MCE, HY-14799), CD3254 (50 nM; MCE, CD3254) and RA (0.5 μM; MCE, HY-14649). Small molecules replacing CHIR 99021 included AZD2858 (2 μM; MCE, HY-15761), LY2090314 (1 μM; MCE, HY-16294), BIO (2 μM; MCE, HY-10580), CHIR 98014 (2 μM; MCE, HY-13076), and SB216763 (10 μM; MCE, HY-216763). To inhibit RAR signaling during culturing of TPS cells, RARγ inhibitor LY2955303 (1 μm; MCE, HY-107765), RARα/β inhibitor LE135 (2 μm; MCE, HY-107436), and RXR inhibitor UVI3003 (1 μm; MCE, HY-107500) were individually added into the CPEC medium. To inhibit LIF/STAT3 signaling during culturing of TPS cells, AG490 (10 μM; MCE, HY-12000), Niclosamide (1 μM; MCE, HY-B0497), JAK inhibitor (10 μM; Millipore, 420097) were individually added into the CPEC medium. After 4–6 passages, cells were collected for further analysis.

### Bulk RNA-seq analysis

Total RNA was isolated from mouse TPS and EPS cells using the RNeasy Mini Kit (Qiagen, 74106). RNA-seq libraries were constructed by using magnetic beads with oligo (dT) enriched mRNA. After that, fragment buffer was added to break the mRNA into short segments. Using mRNA as template, a strand of cDNA was synthesized using six base random primers. Then buffer, dNTPs, DNA polymer I and RNase H were added to synthesize two-strand cDNAs, which were purified using AMPURE XP beads. The purified double-stranded cDNAs were repaired, A-tailed and sequenced, and then the fragment size was selected by AMPURE XP beads. Finally, PCR amplification was carried out and PCR products were purified with AMPURE XP beads to obtain the final library. The fragmented and randomly primed 2× 100-bp paired-end libraries were sequenced using an Illumina HiSeq 2500. The sequencing reads generated were mapped against mouse genome build GRCm38.p6 for mouse using STAR v2.7.3a. The read counts for each gene were calculated, and the expression values of each gene were normalized using TPM. Hierarchical clustering analysis was performed using the PCA function from the package FactoMineR and the ward.D algorithm in R software. DEG analysis was performed using DESeq2 and filtered by adjusted *P* < 0.05. GO analysis was performed using the package topGO and org.Mm.eg.db. Single-cell RNA-seq data of mouse preimplantation embryos (GSE45719^20^) was reanalyzed and the original counts were normalized using TPM. The TPM matrix was used for calculating the average expressions of DEGs between TPS and EPS cells in the preimplantation embryos at different developmental stages. Box plots were used to show the expression of these genes during preimplantation development. To identify totipotent signatures of 2-cell embryos, Seurat objects were constructed using the default parameters from the expression matrix of single-cell RNA-seq data of mouse preimplantation embryos (GSE45719). The top 2000 most variable genes were identified using the ‘vst’ algorithm, and further standardized and normalized for PCA analysis. Genes from the top 11 PCA components were used to construct the KNN map and Uniform Manifold Approximation and Projection (UMAP) map. The epiblast population was identified from the blastocyst cells by the co-expression of Oct4, Nanog and Sox2. DEGs between 2-cell embryos and epiblast cells were identified using FindMarkers (logfc.threshold = 1, min.pct = 0.6, test.use = ‘DESeq2’). A total of 2399 genes were identified as 2-cell embryo enriched genes (*P*adj < 0.0001).

### 10× Genomics single-cell RNA-seq

Cells were washed and resuspended in 1× PBS (calcium and magnesium free) containing 10% FBS. Cell viability was determined by Countstar, and the density of living cells were adjusted to 300–600 living cells/μL. Cell suspension was loaded onto the Chromium single cell controller (10× Genomics). Chromium Single Cell 3ʹ GEM, Library and Gel Bead Kit v3.1 (10× Genomics, 1000075), and Chromium Single Cell B Chip Kit (10× Genomics, 1000074) were used to generate single-cell gel beads in the emulsion according to the manufacturer’s protocol. In short, single cells were suspended in PBS containing 0.04% BSA. About 6000 cells were added to each channel, and the target cells were recovered (~3000 cells). Captured cells were lysed and the released RNAs were barcoded through reverse transcription in individual GEMs. Reverse transcription was performed on a S1000TM Touch Thermal Cycler (Bio-Rad) at 53 °C for 45 min, followed by 85 °C for 5 min, and held at 4 °C. The cDNA was generated and then amplified, and the cDNA quality was assessed using an Agilent 4200 (performed by CapitalBio Technology, Beijing). Single-cell RNA-seq libraries were constructed using Single Cell 3ʹ Library and Gel Bead Kit V3.1 according to the manufacturer’s introduction. The libraries were finally sequenced using an Illumina NovaSeq 6000 sequencer with a sequencing depth of at least 100,000 reads per cell with paired-end 150 bp (PE150) reading strategy (performed by CapitalBio Technology, Beijing).

### 10× Genomics ATAC-seq

Cells were washed and resuspended in 1× PBS (calcium and magnesium free) containing 10% FBS. Cell viability was determined by Countstar. The nuclei were isolated and washed according to the method provided by 10× Genomics: “Nuclei Isolation for Single Cell ATAC Sequencing (CG000169)”. Then the nucleus was resuspended by chilled Diluted Nuclei Buffer (10× Genomics; 2000153). The volume of Diluted Nuclei Buffer used to resuspend nuclei was based on the number of starting cells and the final target nuclei concentration. Countstar (Rigel S2) was used to count the nuclei. The nuclei were then immediately proceeded to construct single-cell ATAC-seq libraries. The nuclei were partitioned into nanoliter-scale GEMs by using Chromium Chip E Single Cell Kit (10× Genomics; 1000156) and Chromium Single Cell ATAC Library & Gel Bead Kit (10× Genomics; 1000110). A pool of ~750,000 10× barcodes was sampled to separately and uniquely index the transposed DNA of each individual nucleus. Then the libraries were generated (performed by CapitalBio Technology, Beijing). The libraries were sequenced using an Illumina NovaSeq sequencer with a sequencing depth of at least 25k read pairs per nucleus with paired-end 50 bp (PE50) reading strategy.

### WGBS

Genomic DNAs were extracted using DNeasy Blood and Tissue Kit (Qiagen, 69504). 100 ng genomic DNAs were fragmented into ~200 bp by Covaris. Then DNAs were bisulfite-converted using EZ DNA Methylation-GoldTM Kit (Zymo, D5005). Bisulfite-converted DNA was captured using Accel-NGS Methyl-Seq DNA Library Kit (Swift Biosciences, 30024). Library samples were sequenced by Illumina NovaSeq 6000 sequencer.

### Single-cell RNA-seq preprocessing

The single-cell RNA-seq data were collected and mapped to mouse reference genome mm10 using Cell Ranger 3.1.0 for all our samples. We performed preprocessing using Seurat 3.2.3^48^ in R environment version 3.6.3. In detail, quality control was firstly performed to remove the cells with (1) total UMI counts < 2000, (2) detected gene number < 1500, or (3) mitochondrial UMI counts > 20%. The normalization was performed using function NormalizeData with default parameters. We performed the standard Seurat clustering pipeline using the following functions: FindVariableFeatures with 2000 genes, ScaleData, RunPCA, FindNeighbors with first 10 PCs, and FindClusters with resolution 1.2. Then we checked the doublet scores using function doubletCells of R package scran for each sample. The scores were evenly distributed across clusters, indicating that there is no inter-cluster doublet detected, therefore we kept all cells for later analysis. We performed batch effect correction using R package Harmony.^49^ The UMAP dimension reduction was performed with first 10 corrected PCs using function RunUMAP. The DEGs were analyzed using function FindMarkers based on normalized gene expression.

### Transcriptional integration analysis of embryo development states and different stem cells

#### Public dataset collection

We collected 12 public datasets to construct a mouse embryonic development trajectory as reference (GSE45719,^20^ GSE109071,^50^ GSE44183,^51^ GSE71434,^52^ GSE66582,^53^ GSE70605,^54^ GSE100597,^55^ GSE84892,^56^ DRP005519,^57^ GSE121708,^58^ GSE136714,^59^ and GSE145609^19^). From these datasets, we used cells from zygote to E7.5 EPI, together with extraembryonic lineages. The cells of epiblast lineage after embryonic day 5 were defined as post-implantation epiblasts.

We also collected 4 stem cell datasets for comparison with our data (GSE33923,^14^ GSE168728,^21^ GSE74155,^53^ and GSE145609^19^). From these datasets, ESCs, EPSCs, Primed EpiSCs, 2CLCs, TBLCs were used in later analyses.

Raw sequencing data of all public datasets were downloaded and subjected to preprocessing following the same procedures to obtain normalized expression data using the same measurement with our data (logTP10K, log normalized transcripts per 10 thousand).

#### Embryonic development trajectory construction

To integrate multiple datasets together to form a continuous development trajectory, we performed latent semantic indexing (LSI) projection as previously described.^60^ In detail, we used GSE45719 and GSE109071 as ‘stem’ datasets and combined them directly. We chose these two datasets for the reasons: (1) they were both sequenced using Smart-Seq2, (2) they both had enough cell numbers for each development state to identify biological variance along the trajectory, and (3) they were widely used for comparison in previous researches. We performed LSI dimension reduction on cells of these two datasets with top 2000 variable genes. Other 10 datasets were then projected to the LSI space. The coordinates of all cells in LSI space were only used for visualization.

#### Stem cell data integration

To compare stem cells from multiple datasets with embryonic development stages, we projected the stem cell data to the LSI space mentioned above. Due to the low sequencing depth of 10× platform for each cell, we combined the cells in our data and GSE168728 to pseudo-bulk samples. The first two LSI dimensions of stem cell samples and cells of epiblast lineage were visualized. The cells of the same development stage were represented as one point at the median of their coordinates.

#### Calculation of embryonic development stage signatures

We calculated gene signature for each embryonic development stage by combing multiple DEG fold change values to a comprehensive score for each gene using weighted average. The DEGs were calculated using DESeq2^61^ for multiple datasets, respectively.

#### ssGSEA

We performed ssGSEA to quantify the similarity between stem cell samples and embryonic development stages using R package GSVA. As control, cells along the embryonic development trajectory were combined and subjected to the same analysis. The enrichment score for each signature was further normalized by dividing the value that positive control can reach for better visualization.

#### Embryonic stage identity analysis

To evaluate the degree of similarity between stem cell samples and given embryonic stages, we performed a quadratic programming-based deconvolution analysis using the R package quadprog as previously described.^23^

### Transcriptional integration analysis of blastocysts and blastoids

We constructed a blastocyst development trajectory using the cells after E3.5 subset from the whole embryonic development trajectory using the same procedure as mentioned above. Our blastoid data were collected and preprocessed in the same way as described above. Two public datasets were collected for comparison (GSE99786^28^ and GSE134240^29^). The LSI projection, ssGSEA, and identity score analysis were performed as described above.

### Single-cell gene regulatory network analysis

We performed SCENIC^62^ analysis on our data following the standard pySCENIC pipeline (https://pyscenic.readthedocs.io/en/latest/index.html). The regulon activities for cells in other datasets were calculated using R package AUCell.

### Single-cell ATAC-seq analysis

The single-cell ATAC-seq data were collected and mapped to mouse reference genome mm10 using Cell Ranger ATAC 1.2.0 for all our samples. The downstream analyses were performed following the standard ArchR^63^ pipeline (https://www.archrproject.com). We collected public ATAC-seq data of mouse preimplantation embryos (GSE66581^53^) for comparison analysis.

### WGBS analysis

We followed the standard Bismark^64^ workflow to perform mapping, deduplication, and methylation extraction (https://github.com/FelixKrueger/Bismark) for all our samples. Only CpG sites were used for further analysis and visualization. We collected public methylome data of mouse embryos (GSE56697^65^) for comparison analysis.

## Supplementary information

Supplementary information, Figure S1

Supplementary information, Figure S2

Supplementary information, Figure S3

Supplementary information, Figure S4

Supplementary information, Figure S5

Supplementary information, Figure S6

Supplementary information, Figure S7

Supplementary information, Figure S8

Supplementary information, Figure S9

Supplementary information, Figure S10

Supplementary information, Table S1

Supplementary information, Table S2

Supplementary information, Table S3

## Data Availability

The RNA-seq, WGBS, single-cell RNA-seq and single-cell ATAC-seq data generated during this study are available at GEO: GSE183522. Additional data are available upon request.
