# Supplementary material for: Derivation of totipotent-like stem cells with blastocyst-like structure forming potential
Source: Cell Res. 2022 May 4;32(6):513–29. doi: 10.1038/s41422-022-00668-0 (PMC9160264; doi:10.1038/s41422-022-00668-0)
Supplement: Supplementary file 1 — Supplementary information, Figure S1 [file 41422_2022_668_MOESM1_ESM.pdf]

Supplementary Figure 1

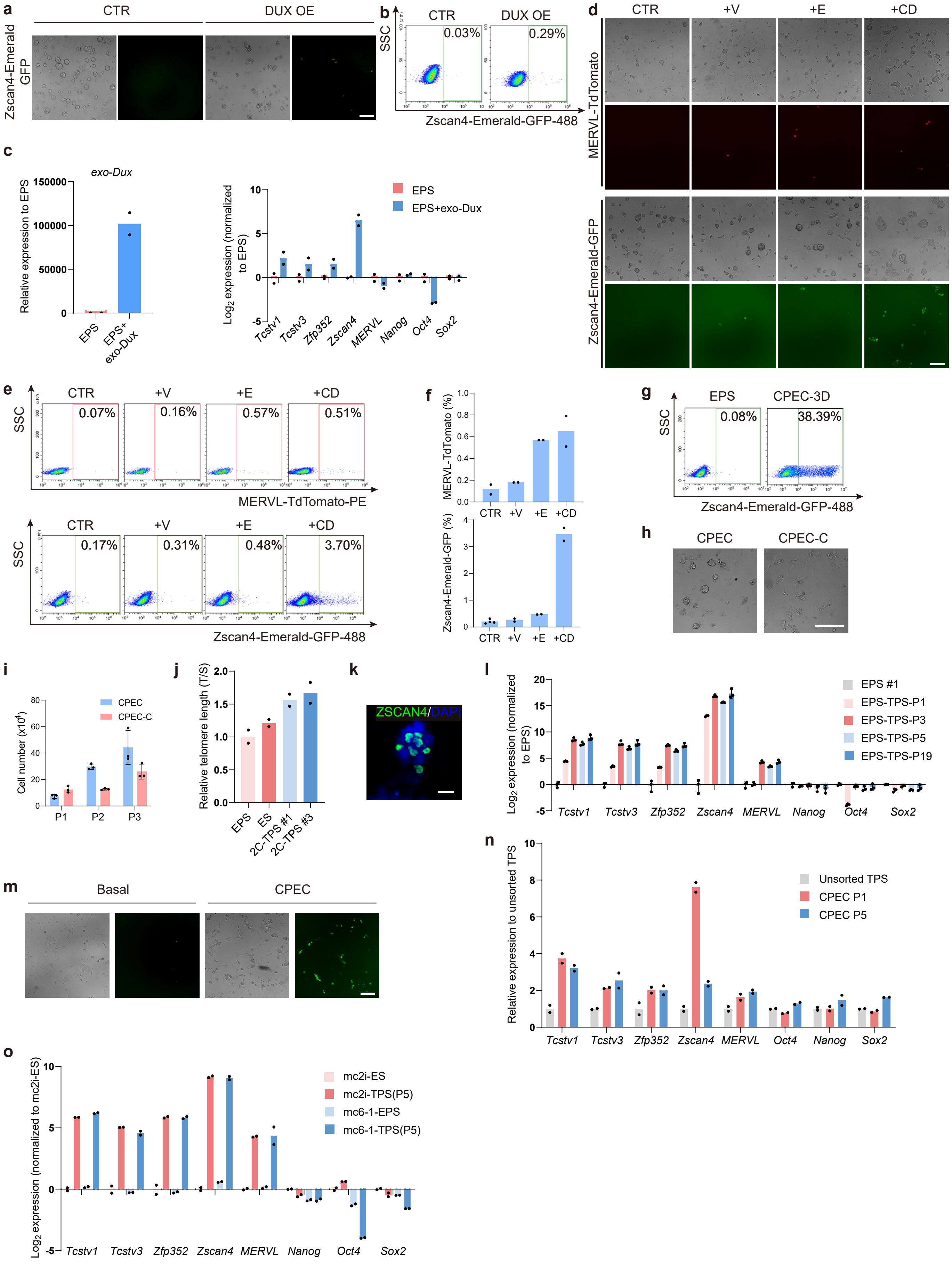

Figure S1. Identification of small molecules that can increase the percentage of cells expressing totipotency marker genes.

a. Representative images showing the effect of exogenous *Dux* transient overexpression on activating Zscan4 reporter expression in EPS cells on day 3. Scale bar, 200 μm. CTR, control. DUX OE, exogenous *Dux* overexpression.

b. Representative flow cytometry analysis of the effect of exogenous *Dux* transient overexpression on activating Zscan4 reporter expression in EPS cells on day 3. The percentage of Zscan4-Emerald-GFP positive cells was shown. CTR, control. DUX OE, exogenous *Dux* overexpression.

c. Q-PCR analysis of expression of exogenous *Dux* (left panel) and totipotency and pluripotency marker genes (right panel) upon exogenous transient overexpression of *Dux*. exo-*Dux*, exogenous *Dux* overexpression. N = 2 biological replicates.

d. Representative images showing the effect of VPA, EPZ004777, and CD1530 on activating Zscan4 or MERVL reporter expression in EPS cells. Scale bar, 200 μm. V, VPA. E, EPZ004777. CD, CD1530. CTR, N2B27 medium supplemented with CHIR 99021.

e-g. Representative flow cytometry analysis of the percentage of Zscan4 or MERVL positive cells in EPS cells on day 3 after treatment of small molecules. For (f), N = 2 biological replicates. V, VPA. E, EPZ004777. CD, CD1530. CTR, N2B27 medium supplemented with CHIR 99021. EPS, EPS cells. CPEC-3D: EPS cells treated with 4 small molecules (VPA, CHIR 99021, EPZ004777, CD1530) for 3 days.

h. Representative images showing the effect of CHIR 99021 on cell proliferation during inducing TPS cells. Scale bar, 200 μm. CPEC-C, CPEC condition without CHIR 99021.

i. Cell number analysis showing that CHIR 99021 promote cell proliferation during the conversion of TPS cells. N = 3 biological replicates. CPEC-C, CPEC condition without CHIR 99021.

j. Q-PCR analysis of telomere length in TPS, ES and TPS cells. N = 2 biological replicates.

k. Representative immunofluorescent analysis of ZSCAN4 expression in TPS cells. Scale bar, 200 μm.

l. Q-PCR analysis of the expression of totipotency and pluripotency marker genes during inducing TPS cells from EPS cells at different passages. EPS, EPS cells. EPS-TPS-P1/3/5/19: converted cells at passage 1/3/5/19. N = 3 biological replicates.

m. Representative images showing sorted Zscan4-Emerald GFP positive cells cultured using the basal medium and CPEC-medium. Scale bar, 200 μm. Basal, basal medium of CPEC condition.

n. Q-PCR analysis of expression levels of representative totipotency and pluripotency marker genes in sorted Zscan4 positive cells cultured in CPEC-medium at passage 1 and 5 (CPEC-P1, CPEC-P5). N = 2 technical replicates. Similar results were obtained in at least 2 independent experiments.

o. Q-PCR analysis of expression levels of representative totipotency and pluripotency marker genes in TPS cells converted from 2i/LIF-ES (mc2i-ES) and LCDM-EPS (mc6-1-EPS) respectively. N = 2 technical replicates. Similar results were obtained in at least 2 independent experiments.
