# Supplementary material for: Derivation of totipotent-like stem cells with blastocyst-like structure forming potential
Source: Cell Res. 2022 May 4;32(6):513–29. doi: 10.1038/s41422-022-00668-0 (PMC9160264; doi:10.1038/s41422-022-00668-0)
Supplement: Supplementary file 2 — Supplementary information, Figure S2 [file 41422_2022_668_MOESM2_ESM.pdf]

Supplementary Figure 2

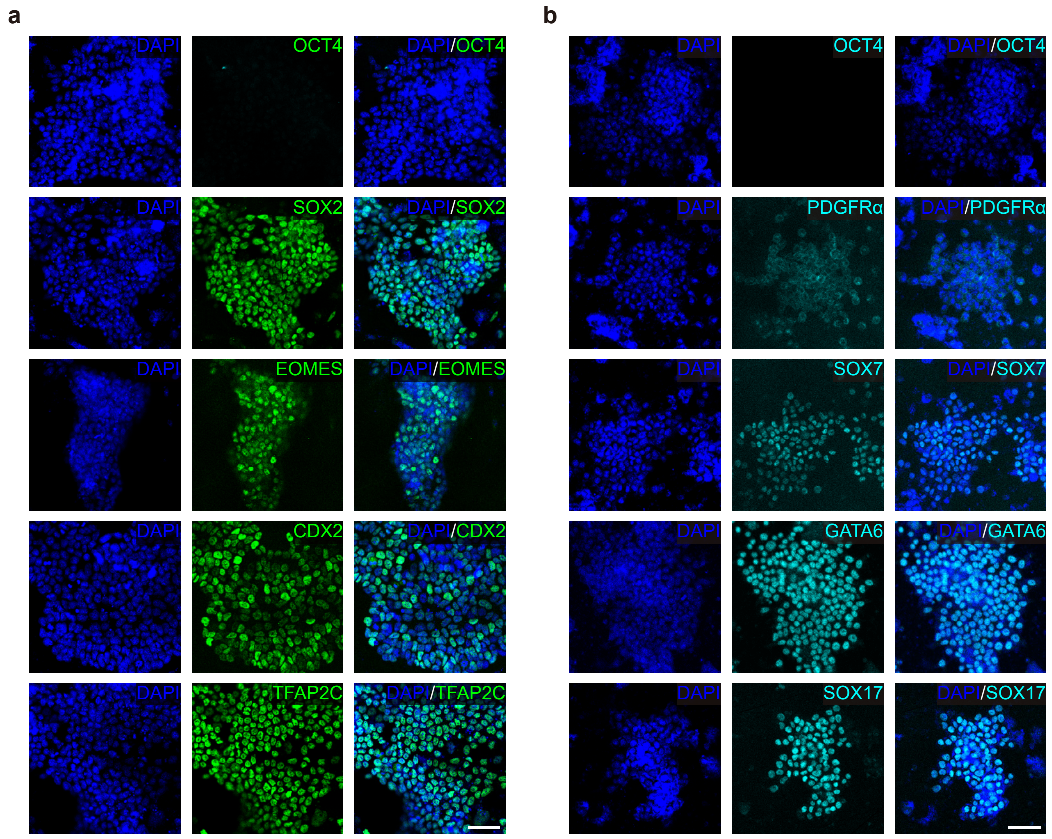

Figure S2. ***In vitro*** **analysis of extraembryonic developmental potentials of TPS cells.**
a. Representative immunofluorescent analysis showing the expression of TS markers (CDX2, EOMES, TFAP2C, SOX2) and pluripotency marker OCT4 in TS-like cells. Scale bar, 50 μm.
b. Representative immunofluorescent analysis showing the expression of PE markers (GATA6, PDGFRα, SOX7, SOX17) and pluripotency marker OCT4 in PE-like cells. Scale bar, 50 μm.
