# Supplementary material for: Derivation of totipotent-like stem cells with blastocyst-like structure forming potential
Source: Cell Res. 2022 May 4;32(6):513–29. doi: 10.1038/s41422-022-00668-0 (PMC9160264; doi:10.1038/s41422-022-00668-0)
Supplement: Supplementary file 3 — Supplementary information, Figure S3 [file 41422_2022_668_MOESM3_ESM.pdf]

Supplementary Figure 3

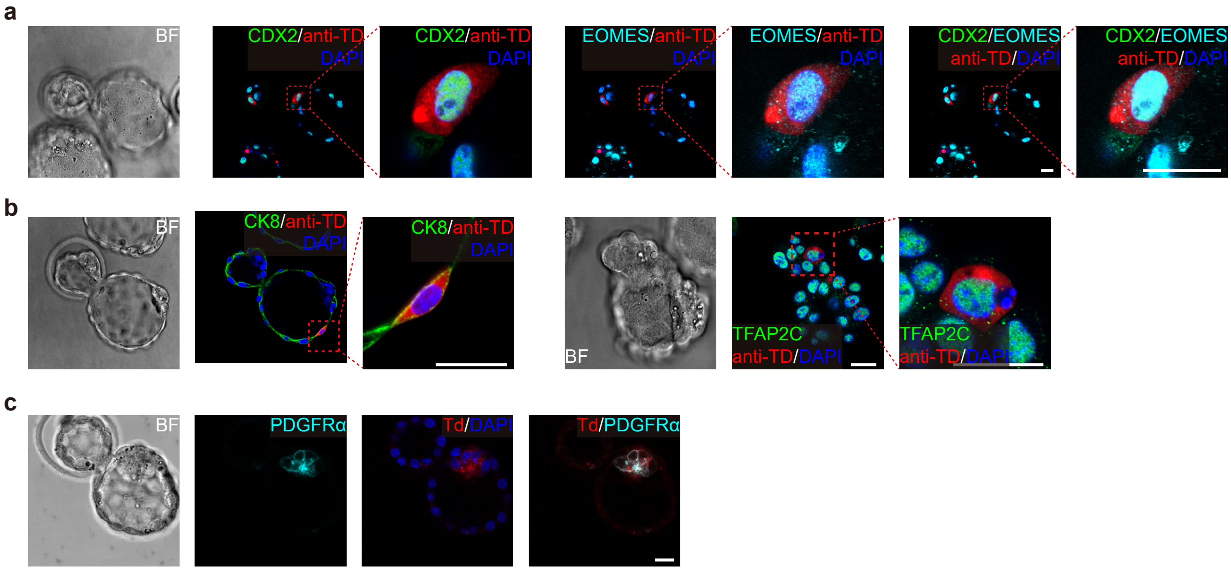

Figure S3. Immunofluorescent analysis of blastocysts with chimeric TPS derivatives *in vitro*.

a-c. Representative immunofluorescent images showing contribution of TPS derivatives in trophoectoderm (CDX2, EOMES, CK8, TFAP2C) and primitive endoderm (PDGFRα) in mouse blastocysts. For fluorescent images, the left panels show the original images and the right panels show the enlarged images. BF, Bright field. Td, endogenous tdTomato. anti-TD, immuno-staining of tdTomato protein. Scale bars, 20 μm.
