# Supplementary material for: Derivation of totipotent-like stem cells with blastocyst-like structure forming potential
Source: Cell Res. 2022 May 4;32(6):513–29. doi: 10.1038/s41422-022-00668-0 (PMC9160264; doi:10.1038/s41422-022-00668-0)
Supplement: Supplementary file 4 — Supplementary information, Figure S4 [file 41422_2022_668_MOESM4_ESM.pdf]

Supplementary Figure 4

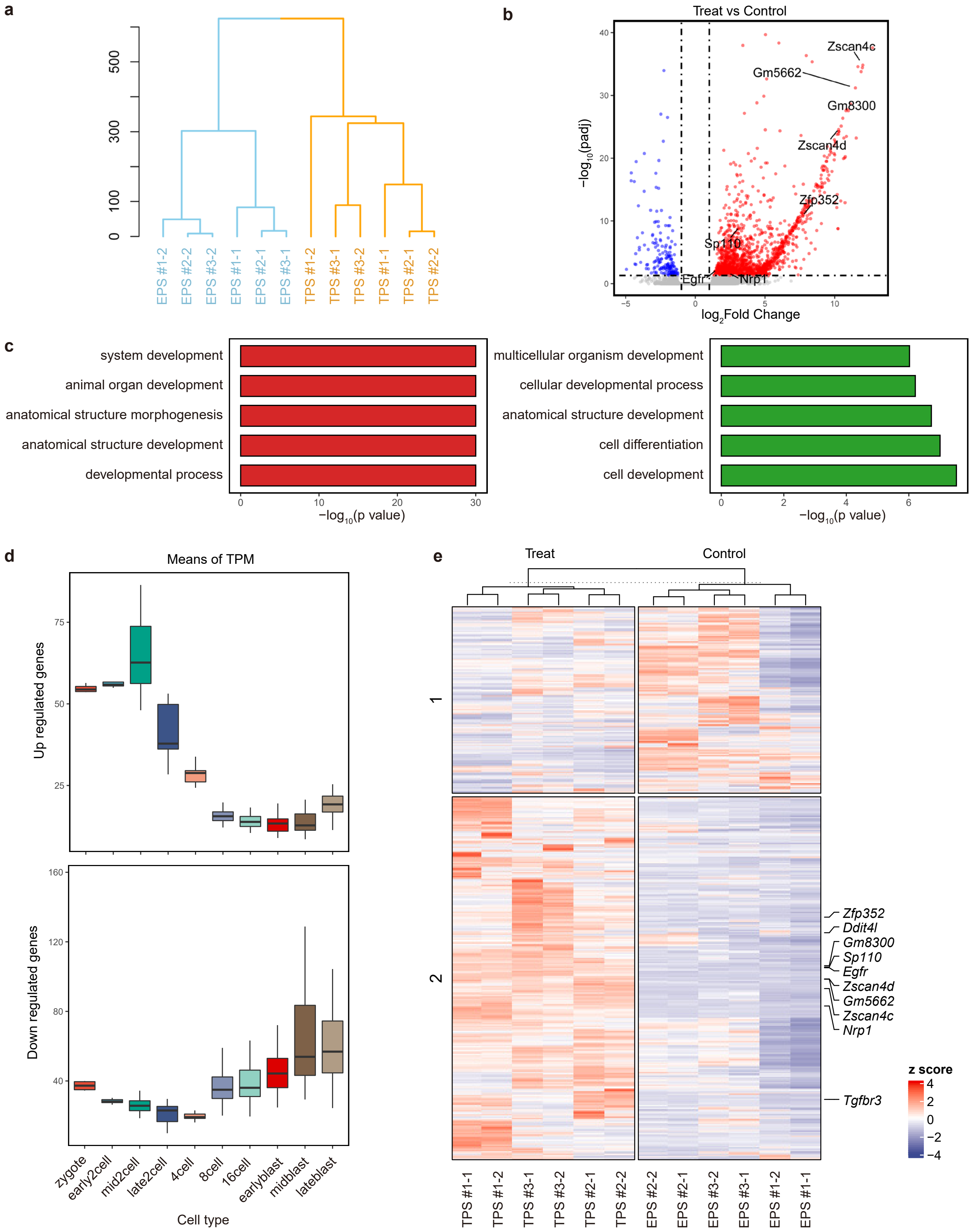

Figure S4. Transcriptomic analysis of TPS cells with 2-cell blastomeres and EPS cells.

a. Hierarchical clustering analysis of TPS and EPS cell lines.

b. Volcano plot showing comparison of transcriptomic differences between TPS and EPS cells. Representative upregulated genes are shown in red plots.

c. Gene Ontology analysis of differently expressed genes between TPS and EPS cells. Left panel, genes upregulated in TPS cells. Right panel, genes downregulated in TPS cells.

d. Box plots showing the expression of genes upregulated and downregulated in TPS cells at different development stages during preimplantation development (Deng et al., 2014).

e. Heatmap showing the expression of 2-cell embryo-specific totipotency signatures in TPS and EPS cells.
