# Supplementary material for: Derivation of totipotent-like stem cells with blastocyst-like structure forming potential
Source: Cell Res. 2022 May 4;32(6):513–29. doi: 10.1038/s41422-022-00668-0 (PMC9160264; doi:10.1038/s41422-022-00668-0)
Supplement: Supplementary file 5 — Supplementary information, Figure S5 [file 41422_2022_668_MOESM5_ESM.pdf]

Supplementary Figure 5

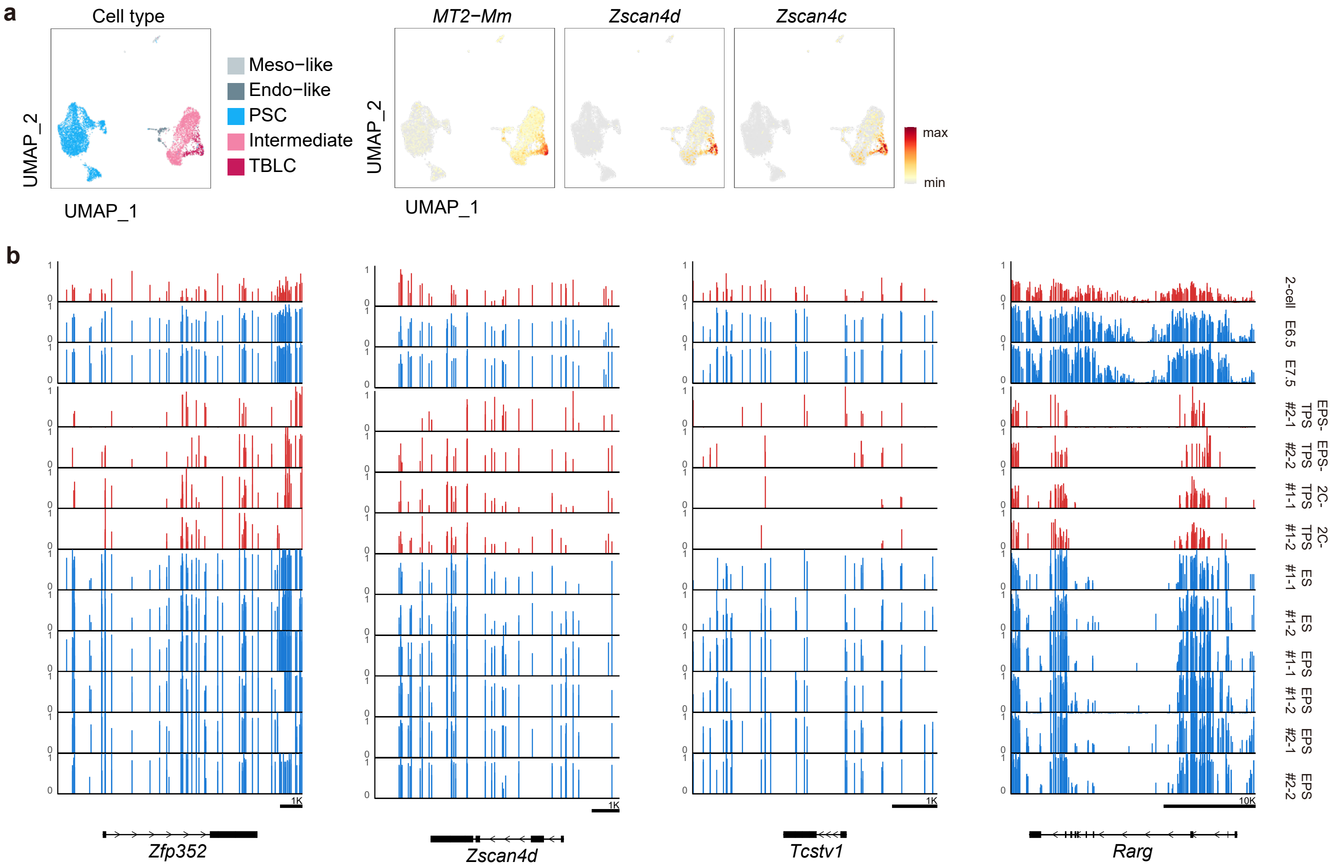

Figure S5. Further analysis of molecular features of TPS cells.

a. UMAP plot showing the expression of representative totipotency marker genes in TBLCs at the single cell level. Different cell types are indicated using different colors. PSC, pluripotent stem cells. Meso-like an Endo-like, fibroblast feeders. Intermediate, intermediate cells in the TBLCs. TBLC, TBLC subpopulation highly expressing totipotency marker genes.

b. WGBS analysis of CpG methylation in the loci of representative totipotency marker genes in 2-cell embryo (2-cell), E6.5 and E7.5 embryos (E6.5 and E7.5), EPS cells, TPS cells, and ES cells.
