# Supplementary material for: Derivation of totipotent-like stem cells with blastocyst-like structure forming potential
Source: Cell Res. 2022 May 4;32(6):513–29. doi: 10.1038/s41422-022-00668-0 (PMC9160264; doi:10.1038/s41422-022-00668-0)
Supplement: Supplementary file 6 — Supplementary information, Figure S6 [file 41422_2022_668_MOESM6_ESM.pdf]

Supplementary Figure 6

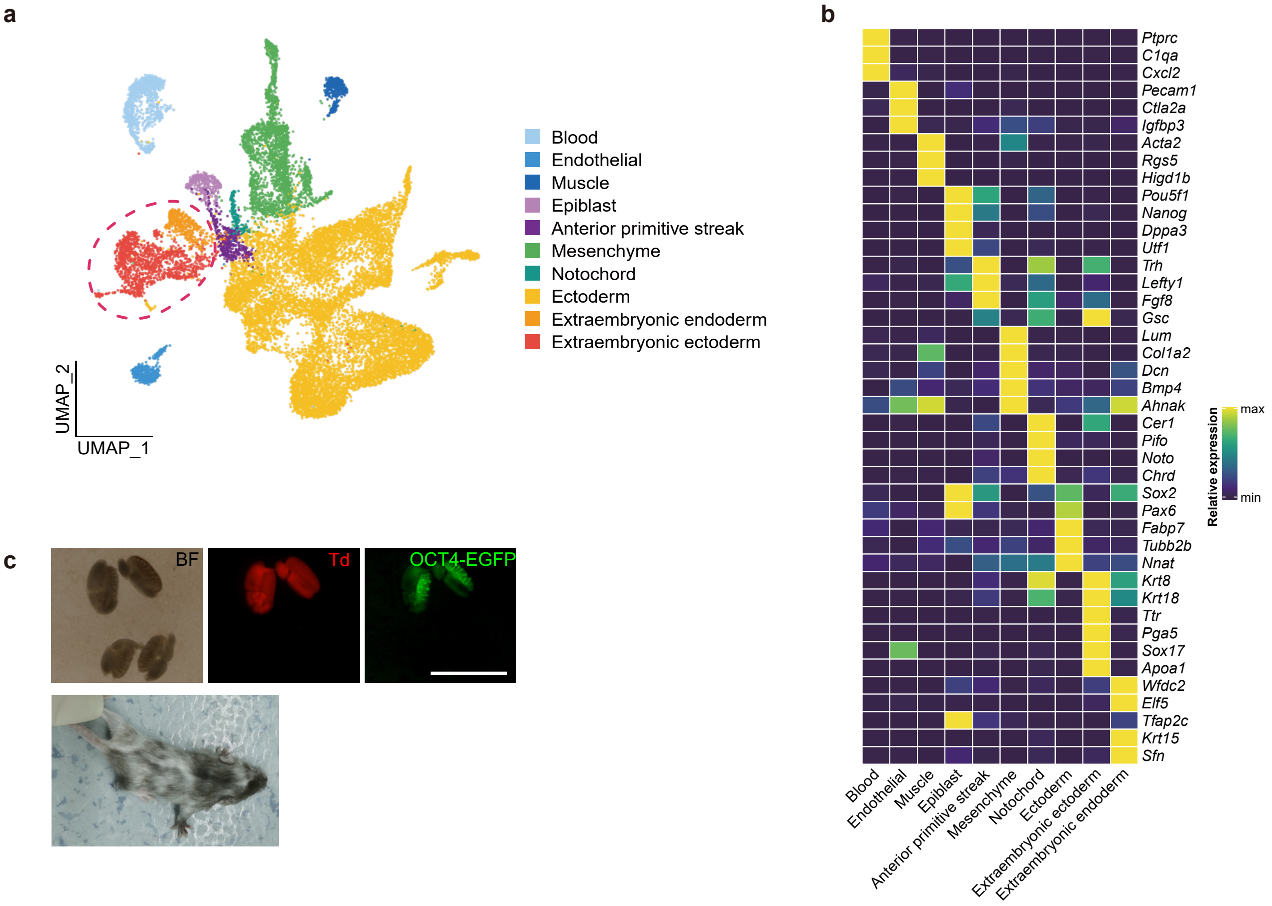

Figure S6. Analysis of the *in vivo* developmental potentials of TPS cells.

a. UMAP plot showing the main clusters in the TPS-derived teratomas. Red dotted line indicates extraembryonic cell lineages.

b. Heatmap showing the average expression of representative marker genes for each cluster from the TPS-derived teratomas.

c. Representative images showing chimerism of TPS derivatives in genital ridge of E13.5 embryos (upper panels), and TPS-derived chimeric mice (lower panel). For upper panels, samples on the upper side were from one chimeric conceptus, and samples on the lower side were from one non-chimeric conceptus. Td, endogenous tdTomato. OCT4-EGFP, *Oct4* promoter driven EGFP. Scale bar, 2 mm. Similar images were obtained in at least 2 independent experiments.
