# Supplementary material for: Derivation of totipotent-like stem cells with blastocyst-like structure forming potential
Source: Cell Res. 2022 May 4;32(6):513–29. doi: 10.1038/s41422-022-00668-0 (PMC9160264; doi:10.1038/s41422-022-00668-0)
Supplement: Supplementary file 7 — Supplementary information, Figure S7 [file 41422_2022_668_MOESM7_ESM.pdf]

Supplementary Figure 7

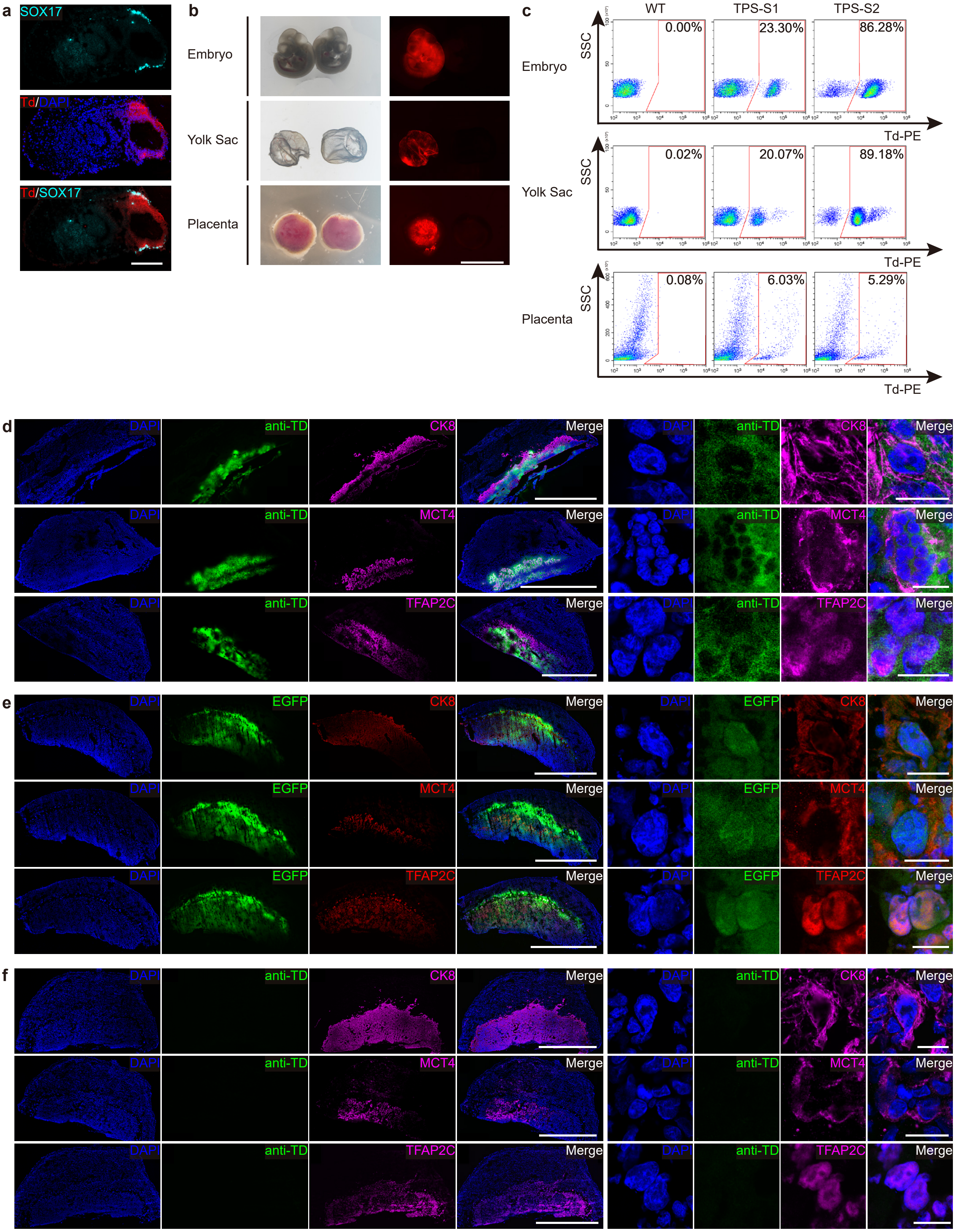

Figure S7. Further analysis of the chimerism of TPS cells *in vivo*.

a. Representative immunofluorescent analysis showing the expression of visceral endoderm marker SOX17 in single TPS derivative cells in E7.5 embryos. Td, endogenous tdTomato. Scale bar, 200 μm. Similar images were obtained in at least 3 independent experiments.

b. Representative images showing contribution of single blastomere of 8-cell embryo continuously expressing tdTomato in E10.5 embryo, yolk sac and placenta. For each image, samples on the left side were from one chimeric conceptus, and samples on the right side were from one non-chimeric conceptus. Scale bars, 5 mm. Similar images were obtained in at least 3 independent experiments.

c. Representative flow cytometry analysis of the chimerism of single TPS derivative cells in E10.5 embryo, yolk sac and placenta. Td, endogenous tdTomato. WT, wild type. Similar images were obtained in at least 3 independent experiments.

d. Representative immunofluorescent analysis of E10.5 chimeric placenta generated by injection of single TPS cell (tdTomato labeled) converted from EPS cells. anti-TD, immuno-staining of tdTomato protein. The right panels show enlarged images of the left panels. Scale bars: left panels, 2 mm; right panels, 20 μm. Similar images were obtained in at least 3 independent experiments.

e. Representative immunofluorescent analysis of E10.5 chimeric placenta generated by injection of single blastomere of 8-cell embryo continuously expressing EGFP. EGFP, endogenous EGFP. The right panels show enlarged images of the left panels. Scale bars: left panels, 2 mm; right panels, 20 μm. Similar images were obtained in at least 3 independent experiments.

f. Representative immunofluorescent analysis of wild type E10.5 placenta. anti-TD, immuno-staining of tdTomato protein. The right panels show enlarged images of the left panels. Scale bars: left panels, 2 mm; right panels, 20 μm. Similar images were obtained in at least 3 independent experiments.
