# Supplementary material for: Derivation of totipotent-like stem cells with blastocyst-like structure forming potential
Source: Cell Res. 2022 May 4;32(6):513–29. doi: 10.1038/s41422-022-00668-0 (PMC9160264; doi:10.1038/s41422-022-00668-0)
Supplement: Supplementary file 8 — Supplementary information, Figure S8 [file 41422_2022_668_MOESM8_ESM.pdf]

Supplementary Figure 8

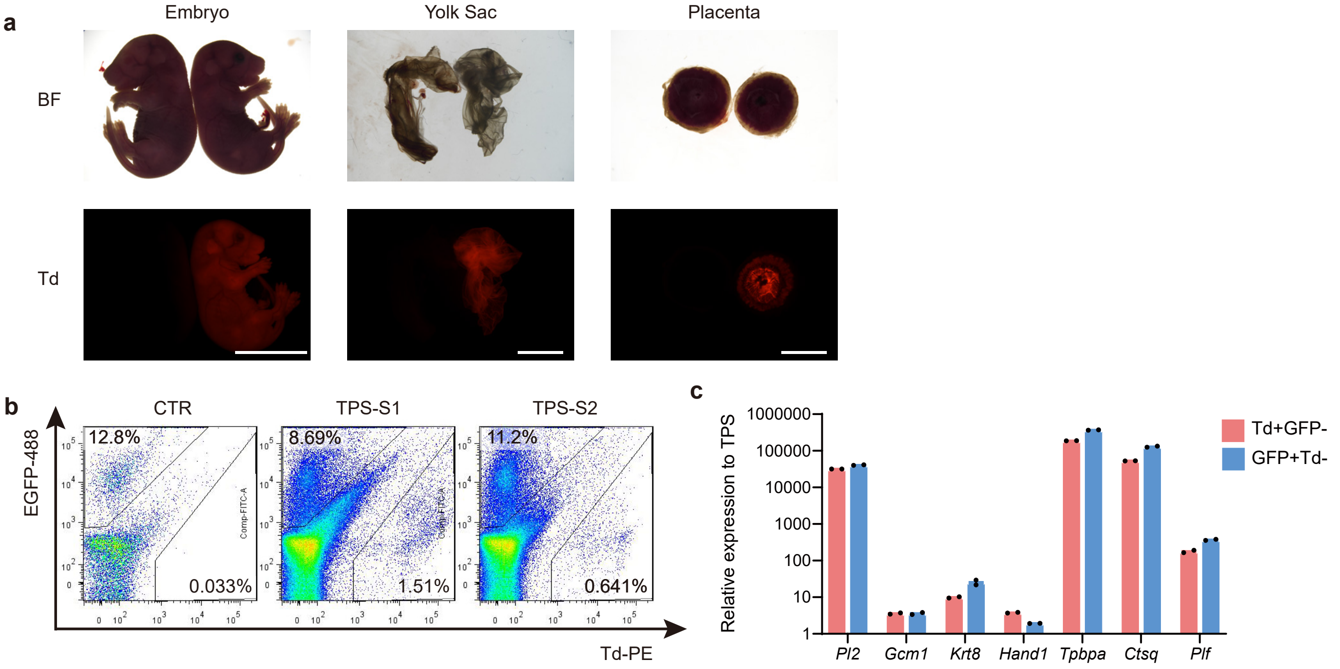

Figure S8. Further analysis of the chimerism in E17.5 conceptuses.

a. Representative images showing contribution of single TPS-derived cells (tdTomato labeled) into embryo, yolk sac and placenta in E17.5 mouse conceptuses. BF: bright field. Td, endogenous tdTomato. For each image, samples on the left side were from one non-chimeric conceptus, and samples on the right side were from one chimeric conceptus. Scale bars, 10 mm. Similar images were obtained in at least 3 independent experiments.

b. Representative flow cytometry analysis of the chimerism of tdTomato positive TPS-derived cells in EGFP positive recipient E10.5 placenta. CTR, control. EGFP-488, endogenous EGFP. Td-PE, endogenous tdTomato. Similar images were obtained in at least 2 independent experiments.

c. Representative Q-PCR analysis of trophoblast marker genes expression in tdTomato positive TPS-derived cells in EGFP positive recipient E18.5 placenta tissues. N = 2 technical replicates. tdTomato positive and EGFP negative cells (Td+GFP-) were purified by FACS. GFP positive and tdTomato negative cells (GFP+Td-) were also sorted as the control. Similar results were obtained in at least 2 independent experiments.
