# Supplementary material for: Derivation of totipotent-like stem cells with blastocyst-like structure forming potential
Source: Cell Res. 2022 May 4;32(6):513–29. doi: 10.1038/s41422-022-00668-0 (PMC9160264; doi:10.1038/s41422-022-00668-0)
Supplement: Supplementary file 9 — Supplementary information, Figure S9 [file 41422_2022_668_MOESM9_ESM.pdf]

Supplementary Figure 9

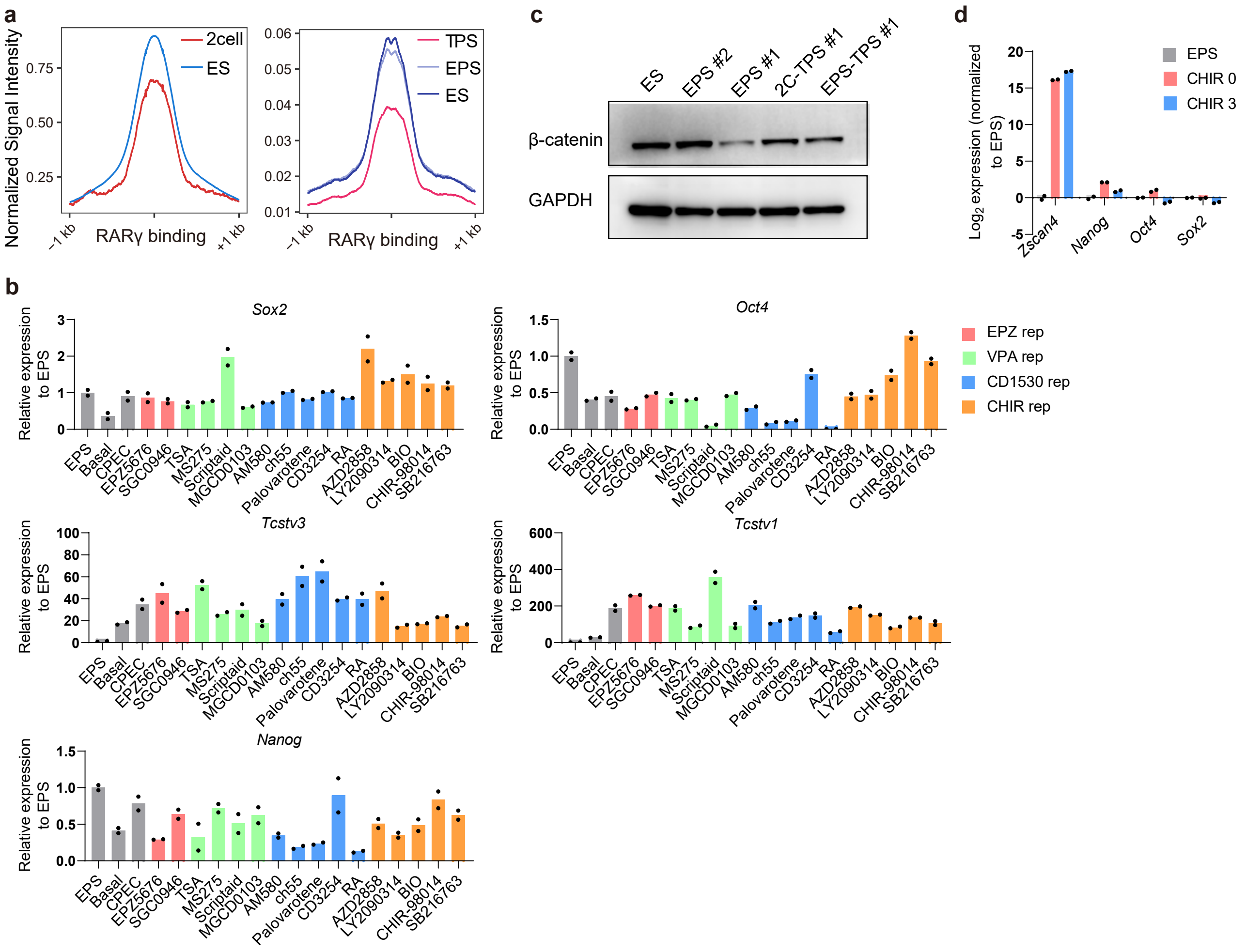

Figure S9. Further mechanistic exploration of totipotency induction and maintenance in TPS cells.

a. Average ATAC-seq signal intensities of genomic regions containing RARγ motif in TPS, ES, EPS cells and 2-cell embryos (2cell). "ES" in the right image indicates sequencing data of ES cell samples that were collected and sequenced in this study. "ES" in the left image indicates sequencing data of ES cells from public resources.

b. Q-PCR analysis of expression levels of representative totipotency and pluripotency marker genes on day 3 upon treatment of different small molecules combinations. In the CPEC condition, EPZ004777, VPA, CD1530 and CHIR 99021 were replaced by small molecules target DOT1L, HDAC, RA signaling and GSK3β respectively. EPS, EPS cells. Basal, EPS cells cultured in the basal medium of CPEC condition. EPZ rep, VPA rep, CD1530 rep and CHIR rep indicate small molecules that target DOT1L, HDAC, RA and GSK3β respectively. N = 2 technical replicates. Similar results were obtained in at least 2 independent experiments.

c. Western blot analysis showing the protein expression of β-catenin in ES, EPS and TPS cells. ES, ES cells. EPS, EPS cells. 2C-TPS, TPS cells derived from 2-cell embryos. EPS-TPS, TPS cells converted from EPS cells.

d. Q-PCR analysis of expression levels of totipotency and pluripotency marker genes in TPS cells cultured in CHIR 0 and CHIR 3 condition. CHIR 0 ,CPEC condition without CHIR 99021; CHIR 3, CPEC condition. N = 2 technical replicates. Similar results were obtained in at least 2 independent experiments.
