# Supplementary material for: Derivation of totipotent-like stem cells with blastocyst-like structure forming potential
Source: Cell Res. 2022 May 4;32(6):513–29. doi: 10.1038/s41422-022-00668-0 (PMC9160264; doi:10.1038/s41422-022-00668-0)
Supplement: Supplementary file 10 — Supplementary information, Figure S10 [file 41422_2022_668_MOESM10_ESM.pdf]

Supplementary Figure 10

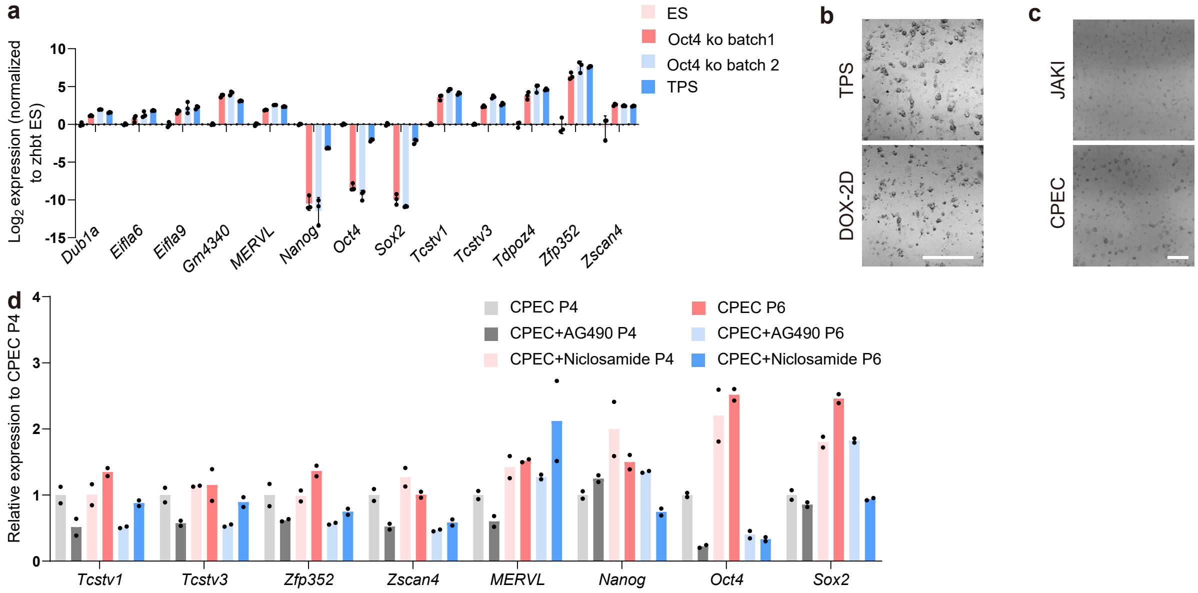

Figure S10. Analysis of the role of Oct4 and LIF/STAT3 signaling in regulating TPS cells proliferation and totipotency markers expression.

a. Q-PCR analysis showing the effect of *Oct4* knockout on expression levels of representative totipotency and pluripotency marker genes in TPS cells. Oct4 ko batch 1 and batch 2 indicate different batches of experiments. N = 3 biological replicates.

b. Representative images showing the effect of *Oct4* knockout on TPS cell proliferation. Scale bar, 500 μm. DOX-2D, 2 days after addition of doxorubicin.

c. Representative images showing the effect of inhibition of LIF signaling on the proliferation of TPS cells. JAK inhibitor (JAKI) was used. Scale bar, 500 μm. Similar results were obtained in at least 2 independent experiments.

d. Q-PCR analysis of expression levels of representative totipotency and pluripotency marker genes in TPS cells upon inhibition of LIF signaling by Jak inhibitors AG490 and Niclosamide. Cells at passage 4 and 6 were analyzed. N = 2 technical replicates. Similar results were obtained in at least 2 independent experiments.
