# Supplementary material for: Derivation of totipotent-like stem cells with blastocyst-like structure forming potential
Source: Cell Res. 2022 May 4;32(6):513–29. doi: 10.1038/s41422-022-00668-0 (PMC9160264; doi:10.1038/s41422-022-00668-0)
Supplement: Supplementary file 11 — Supplementary information, Table S1 [file 41422_2022_668_MOESM11_ESM.pdf]

Table S1: Chimeric summary

Summary of 8-cell embryo chimeric assay by injecting TPS cells from different passages				
Detected stage	Passage	Recovered embryos	Contributed into both emb&extraemb	injected cell number (cells/number)
5.5	P3	11	1	10~15
5.5	P4	7	1	10~15
7.5	P4	11	0	1
10.5	P5	10	3	1
10.5	P6	9	2	1
10.5	P8	20	8	1

Summary of chimeric assay by injection of a single cell into 8-cell embryo derived from E7.5			
Cell lines	Injected embryos	Recovered embryos	Contributed into both emb&extraemb
TPS #1	44	26	6
TPS #2	49	18	6
TPS #3	48	15	3

Summary of chimeric assay by injection of a single cell into 8-cell embryo derived from E10.5			
Cell lines	Injected embryos	Recovered embryos	Contributed into both emb&extraemb
TPS #1	65	30	11
TPS #2	66	22	12

Summary of chimeric assay by injection of a single cell into 8-cell embryo without CHIR addition		
Cell lines	Recovered embryos	Contributed into both emb&extraemb
CPEC-C #1	93	3
CPEC-C #2	62	1

Representative analysis of blastoid induction efficiency		
Analyzed structures	Blastocyst-like structures	Blastoid induction efficiency
89	29	32.60%
