# Supplementary material for: Derivation of totipotent-like stem cells with blastocyst-like structure forming potential
Source: Cell Res. 2022 May 4;32(6):513–29. doi: 10.1038/s41422-022-00668-0 (PMC9160264; doi:10.1038/s41422-022-00668-0)
Supplement: Supplementary file 13 — Supplementary information, Table S3 [file 41422_2022_668_MOESM13_ESM.pdf]

Table S3-1: qPCR primers used in this study

Gene	Primer name	Sequence (5' to 3')
MuERV-c	MERVL_c_F	GGTGGTCGAGATGGAGGTTA
	MERVL_c_R	ATGAAGGAAGTGGTCCAACG
MuERV-d	MERVL_d_F	ACAGCCTAATCCAAGCAGGA
	MERVL_d_R	ATCCTCGTTTCTGCAACTGG
Zscan4	Zscan4-1-F	GAGATTCATGGAGAGTCTGACTGATGAGTG
	Zscan4-1-R	GCTGTTGTTTCAAAAGCTTGATGACTTC
Zfp352	ZFP352-1-F	AAGTCCCACATCTGAAGAAACAC
	ZFP352-1-R	GGGTATGAGGATTCACCCACA
Tcstv1	Tcstv1-F	TGAACCCTGATGCCTGCTAAGACT
	Tcstv1-R	AGATGGCTGCAAAGACACAACTGC
Tcstv3	Tcstv3-F	AGAAAGGGCTGGAACTTGTGACCT
	Tcstv3-R	AAAGCTCTTTGAAGCCATGCCCAG
Nanog	Nanog-1-F	TCTTCCTGGTCCCCACAGTTT
	Nanog-1-R	GCAAGAATAGTTCTCGGGATGAA
Oct4	Oct3/4-up	TGAGGAGGGATTAAAAGCACA
	Oct3/4-dn	CAAAATGATGAGTGACAGACAGG
Sox2	mSox2-up	AAACCACCAATCCCATCCA
	mSox2-dn	CCCCAAAAAGAAGTCCCAAG
Cdx2	cdx2-2-F	AAACCTGTGCGAGTGGATG
	cdx2-2-R	TCTGTGTACACCACCCGGTA
Krt8	Krt8-q-F-1	GTCCATCAGGGTGACTCAGAAATC
	Krt8-q-R-1	GAAAAGCTGGAAGAGCTGATGC
β-actin	mActinb_fwd	TAGGCACCAGGGTGTGATGG
	mActinb_rev	CATGGCTGGGGTGTTGAAGG
PL2	q-pl2-F2	CCAACGTGTGATTGTGGTGTC
	q-pl2-R2	CAGGCCATAGGTCCAAGCTG
GCM1	q-gcm1-F2	CCTCCAACTCCTTACGGATGA
	q-gcm1-R2	CCTCCAACTCCTTACGGATGA
Hand1	q-Hand1-F2	CTACCAGTTACATCGCCTACTTG
	q-Hand1-R2	ACCACCATCCGTCTTTTTGAG
Tpbpa	q-Tpbpa-F2	CACAGTAGCGAAAATGACCAGG
	q-Tpbpa-R2	TCCTCCTCTTCAAACATTGGGT
Ctsq	q-Ctsq-F2	CATTGCCAGTTGACAACACAAG
	q-Ctsq-R2	ATAGCCTTCATTTCGCCAATCA
PLF	q-plf-F1	TCCTGGATACTGCTCCTACTACT
	q-plf-R1	GACCATTCCTCATTGCACACA

Dux	Dux-1-F	CCCAGCGACTCAAACTCCTTC
	Dux-1-R	GGACTTCGTCCAGCAGTTGAT
Dub1a	Dub1a-up	GGTCTCAGATCAAGTGTCACCAC
	Dub1a-dn	ATTCTCCCCACATAGCTCTTCTG
Eiflal6	Eif1al6-F	CAACACAATTGGCATCTTGACG
	Eif1al6-R	GCATTGACAAAACACACTCATGC
Eiflal9	Eif1al9-F	CAATGCAGGTAGTTGTTAAGAGCA
	Eif1al9-R	GAAAATGGCTTAACACAAAGGACA
Gm4340	GM4340-up	AATCGAGGCACTGGGTCTAAG
	GM4340-dn	GCCGCCCCTATTTTTGCTTTG
Tdpoz4	Tdpoz4-up	GATGATGGGCTTCATCTACACAG
	Tdpoz4-dn	CTCACACATGACCATCAAGTCCT
exo-Dux	DUX-synthesis-q-F-1	ATCCCGGTAGCATGGAGCAT
	DUX-synthesis-q-R-1	CACTTGTGGGGGTTGTGACTTT
Cebpa	q-Cebpa-F	CAAGAACAGCAACGAGTACCG
	q-Cebpa-R	GTCACTGGTCAACTCCAGCAC
Cyp26a1	q-Cyp26a1-F1	AAGCTCTGGGACCTGTACTGT
	q-Cyp26a1-R1	CTCCGCTGAAGCACCATCT
Hoxa1	q-hoxa1-F2	CCCAGACGGCTACTTACCAGA
	q-hoxa1-R2	CATAAGGCGCACTGAAGTTCT
Hoxa5	q-hoxa5-F	CTCATTTTGCGGTCGCTATCC
	q-hoxa5-R	ATCCATGCCATTGTAGCCGTA
Hoxb1	q-hoxb1-F	GCCCCAACCTCTTTTCCCC
	q-hoxb1-R	GACAGGATACCCCGAGTTTTG
Rarb	q-rarb-F	CTGCTCAATCCATCGAGACAC
	q-rarb-R	CTTGTCCTGGCAAACGAAGC
Stra8	q-Stra8-F1	ACAACCTAAGGAAGGCAGTTTAC
	q-Stra8-R1	GACCTCCTCTAAGCTGTTGGG
Hdac1	Q-Hdac1-F-1	AGTCTGTTACTACTACGACGGG
	Q-Hdac1-R-1	TGAGCAGCAAATTGTGAGTCAT
Hdac2	Q-Hdac2-F-3	CGGATGACTCATAACTTGCTGC
	Q-Hdac2-R-3	TTCTCCGACGTTAAATCTCTGC
Dot1l	Q-Dot1l-F-3	CAACTGCAAACATCACTACGGA
	Q-Dot1l-R-3	TCACCTCGTTCCAGTGTGTAT
p53	p53-F	GTCACAGCACATGACGGAGG
	p53-R	TCTTCCAGATGCTCGGGATAC
Telomere	telomer-F	CGGTTTGTTTGGGTTTGGGTTTGGGTTTGGGTTTGG GTT

Telomere	telomer-R	GGCTTGCCTTACCCTTACCCTTACCCTTACCCTTAC CCT
36B4	36B4-F	ACTGGTCTAGGACCCGAGAAG
	36B4-R	TCAATGGTGCCTCTGGAGATT

Table S3-2: Oligonucleotides used in this study

**Cloning sgRNA in px330**	
Dux sgRNA 1#_fwd	AAGGCACACAGCCGCTTGCT
Dux sgRNA 1#_rev	AGCAAGCGGCTGTGTGCCTT
Dux sgRNA 1#_fwd	GACTTTCCCCACTAGTGGCT
Dux sgRNA 2#_rev	AGCCACTAGTGGGGAAAGTC
p53 sgRNA 1#_fwd	GAACAGATCGTCCATGCAGTG
p53 sgRNA 2#_rev	CACTGCATGGACGATCTGTTC
p53 sgRNA 1#_fwd	GTCCACCCGGATAAGATGCTG
p53 sgRNA 2#_rev	CAGCATCTTATCCGGGTGGAC
**shRNA sequence in TRC1.5 Vector (pLKO.1-puro Vector)**	
Hdac1 shRNA 1#	CCGGGCTTGGGTAATAGCAGCCATTCTCGAGAATGGCTGCTATTACCCAAGCTTTTTG
Hdac1 shRNA 2#	CCGGGCCAGTCATGTCCAAAGTAATCTCGAGATTACTTTGGACATGACTGGCTTTTTG
Hdac1 shRNA 3#	CCGGGCGTTCTATTCGCCCAGATAACTCGAGTTATCTGGGCGAATAGAACGCTTTTTG
Hdac2 shRNA 1#	CCGGCCCAATGAGTTGCCATATAATCTCGAGATTATATGGCAACTCATTGGGTTTTTG
Hdac2 shRNA 2#	CCGGCGAGCATCAGACAAACGGATACTCGAGTATCCGTTTGTCTGATGCTCGTTTTTG
Hdac2 shRNA 3#	CCGGCGATCAATAAGACCAGATAATCTCGAGATTATCTGGTCTTATTGATCGTTTTTG
Dot1l shRNA 1#	CCGGGTCCAGTTTGTACTGTCAATACTCGAGTATTGACAGTACAAACTGGACTTTTTG
Dot1l shRNA 2#	CCGGGCTGACCTACAATGACCTGATCTCGAGATCAGGTCATTGTAGGTCAGCTTTTTG
Dot1l shRNA 3#	CCGGCCTCGGTTTACACAGCTTCAACTCGAGTTGAAGCTGTGTAAACCGAGGTTTTTG
control shRNA	non-target shRNA (SHC002, Smiga-Aldrich)

Table S3-3: Gene sequences used in this study

Gene	Sequence (5' to 3')
exo-DUX	ATGGACTACAAAGACGATGACGACAAGGGATCCACTAGTAACGGCCGCCAGTGTGCTGGAAT<br>TCTGGCTGAAGCTGGGTCTCCTGTGGGAGGCTCCGGAGTTGCCAGAGAGTCTAGAAGGAGGA<br>GGAAGACTGTGTGGCAAGCTTGGCAGGAGCAGGCCTTGTTGAGTACCTTCAAAAAGAAGAGA<br>TATCTCTCTTTTAAGGAAAGGAAGGAGTTGGCCAAAAGGATGGGGGTATCTGACTGCAGGAT<br>CAGAGTATGGTTCCAAAATAGGCGAAACAGATCAGGAGAGGAAGGGCATGCTTCAAAGAGGA<br>GCATAAGAGGATCCAGGCGACTGGCCTCACCCCAGCTCCAGGAAGAATTGGGGAGTAGACCT<br>CAGGGCAGAGGCATGAGATCCAGCGGGAGAAGGCCTAGAACTAGACTGACAAGTCTCCAGCT<br>CAGGATCTTGGGGCAAGCATTTGAGAGAAATCCTAGACCAGGGTTTGCAACCAGGGAGGAGC<br>TTGCTAGGGACACCGGCCTGCCAGAGGACACTATTCATATATGGTTTCAGAATAGAAGGGCTC<br>GAAGAAGGCACAGGAGGGGAAGACCTACCGCCCAGGATCAGGACCTCTTGGCTAGTCAAGGA<br>TCTGATGGTGCACCAGCCGGCCCAGAAGGAAGAGAAAGAGAGGGAGCTCAAGAAAATCTTCT<br>TCCCCAAGAAGAGGCAGGAAGCACAGGAATGGACACATCCTCTCCATCTGATCTTCCTAGCTT<br>TTGTGGTGAATCCCAACCCTTCCAAGTAGCACAACCAAGGGGAGCCGGACAACAAGAAGCAC<br>CTACAAGGGCAGGGAATGCTGGATCCCTCGAACCATTGCTGGACCAGTTGCTTGATGAGGTGC<br>AAGTTGAGGAACCAGCACCTGCACCACTGAACCTGGACGGAGATCCTGGTGGGAGAGTACAT<br>GAAGGGTCCCAAGAGAGCTTCTGGCCACAAGAAGAGGCAGGTTCAACTGGCATGGATACCAG<br>TTCTCCTTCTGATTCTAATTCTTTTTGTAGGGAGAGCCAACCAAGCCAGGTGGCCCAACCTTGT<br>GGTGCTGGGCAGGAGGATGCAAGGACTCAAGCTGATTCAACAGGCCCACTTGAACTGTTGTT<br>GCTGGACCAACTGCTGGATGAAGTTCAGAAAGAAGAGCATGTCCCAGTACCACTTGACTGGG
